# Blood culture practices and microbiological capacity for sepsis diagnostics in Europe (2021–2022): a cross-sectional analysis of the European Sepsis Care Survey

**DOI:** 10.1016/j.lanepe.2025.101570

**Published:** 2025-12-18

**Authors:** Christian S. Scheer, Evangelos J. Giamarellos-Bourboulis, Djillali Annane, Antonio Artigas, Abdullah Tarik Aslan, Gabriella Bottari, Hjalmar R. Bouma, Vladimir Černý, Renata Curić Radivojević, Ken Dewitte, Daniela Filipescu, Matthias Gründling, Johanna Hästbacka, Said Laribi, Annmarie Lassen, Konstantin Lebedinskii, Adam Linder, Jan Máca, Manu L.N.G. Malbrain, Gianpaola Monti, Marlies Ostermann, Michael Osthoff, José Artur Paiva, Michela Sabbatucci, Jakub Śmiechowicz, Mihai Gabriel Ştefan, Marcus Vollmer, Natalija Vuković, Kyriakos Zaragkoulias, Konrad Reinhart, Ricard Ferrer, Evgeny A. Idelevich, European Sepsis Care Study Group on behalf of the ESCMID Study Group for Bloodstream Infections, Endocarditis and Sepsis (ESGBIES) and the European Sepsis Alliance (ESA)av, Alexander Scharf, Adam Leoniuk, Adam Linder, Adrià Albis Guimet, Adriana América Da Silva, Adriana Cataldo, Agata Dąbkowska, Agneverszka Głusek, Aikaterini Ioakeimidou, Alberto del Castillo Blanco, Albertus Beishuizen, Aldona Szczepańska, Alejandro Martin-Quiros, Alejandro Rodriguez, Alessandra Oliva, Alessandro Manfredi, Alessia Franceschi, Alessio Bertini, Alessio Maroccia, Alexander Koch, Alexander Nicko, Alexandre Bleibtreu, Alfonso Arizmendi Demay, Ali Rezaei, Aliaksei Kazachonak, Alicja Bartkowska-Śniatkowska, Almudena Alonso Ojembarrena, Alvise Tosoni, Marco Daverio, Ana Margarida Correia, Ana Maria Munteanu, Anabela Bártolo, Anamaria Harabor, Anani Akpabie, Andrea Carsetti, Andrea Magaard, Andreas Ekman, Andreas Hecker, Andreas Nowak, Andreas Ostermeier, Andreas Prengel, Andreas Sielenkämper, Andreas Thierbach, Andriy Krystopchuk, Andrzej Pihowicz, Aneverk Crombach, Angela Liebke, Anita Lukic, Anja Heymann, Sebastian Baßeler, Anja-Tamara Lange, Anna Antes-żupa, Anna Efthymiou, Anna Eklund, Anna Lukaszewska, Anna Meuronen, Anna Patrizia Poli, Anna Radlińska, Anna Rekas-Dudziak, Anne Floor Heitz, Annelies Tacx, Anni Pulkkinen, Annmarie Lassen, Antonella Frattari, Antonella Viola, Antonija Vrbanić Šutalo, Antonio Carneiro, António Manuel Pereira De Figueiredo, Antti Mäkelä, Arion-Björn Paulus, Armin Sablotzki, Armin Seibel, Arne Röhrs, Athanasios Chalkias, Axel Neverrhaus, B. Anuszkiewicz, Balazs Kadar, Bartolo Michelangelo, Bea Van den Poel, Belinda De Simone, Benjamin Sasko, Bernd Müllejans, Bernhard Birmes, Bernuy Galvez Cesar, Berta Cisteró, Birgit Gottschlich, Biserka Mustac-Hlebec, Björn Jäschke, Boris E. Sakakushev, Bozidar Duplancic, Bruno Van Herendael, Ibrahim Bushra, Anna Mansson, Caitriona Rayner, Calancia Cosmin, Carla Hopper, Carles Triginer, Carlo Pallotto, Carmen Lauterbach, Carmen Rosa Fraga Quintana, Carolina S. Romero, Caroline Riske, Caterina Gentili, Christian Byhahn, Christian Keuneke, Christian Prause, Christian Schlottke, Christina Lydon, Christine Weis, Christoph Büttner, Christoph Dodt, Christoph Peter, Christoph Plank, Christophe Aveline, Cinzia Barletta, Claudia Gutsche, Claudia Holzbauer, Constantin Bodolea, Cordula Thörmer, Johan Courjon, Cristian Cobilinschi, Cristina Honorato, Cristina López Viloria, Csaba Kopitkó, Dagmar Engemann, Dan Corneci, Daniel Bender, Daniela Ionescu, Daniele Orso, Daphne Annika van Rijssel, David Andaluz Ojeda, David Honan, David Míra, Dietmar Dörschner, Dietrich Keller, Dimitrios Schizas, Dimitris Bliamplias, Dirk Hinz, Dirk Meininger, Ditte Gry Strange, Djillali Annane, Astrid Schwurack, Dokoula Carine, Dominik Einwag, Dorel Sandesc, Ilse Van Cotthem, Eefje Verwulgen, Elena Chircov, Eleni Karlafti, Elina Riihioja, Elisabeth Montel, Elora Pavla Matanović, Els-Marie Rolén, Else van den Berg, Erica Sermijn, Ester Rovira Lázaro, Etienne Javouhey, Eva Schaden, Evangelia Michail Michailidou, Eveny Winklaar, Evgeni Dimitrov, Evgeni Nikolaev, Evy Voets, Ewa Zabul, F. Xavier López Altimiras, Fabio Caramelli, Fabrizio DAcapito, Fausto Catena, Ferran Llopis, Filip Abedinov, Filip Triest, Filippia Nikolaou, FIlippo Luciani, Fiona Moore, Simin Aysel Florescu, Florian Berteau, Florian Prätsch, Florian Schache, Florian Seidlitz, Florin Teodor Bobirca, Francesca Greco, Francesca Lelli, Francesco Fleres, Francisco Jose Ezponda, Franco Apra, Frank Fröhlich, Frank Hendrich, Frank Schleibach, Frank Wolffgramm, Frank Zoerner, Frank-Günter Mewes, Frank-R. Klefisch, Fredrik Schiöler, Friedrich Afflerbach, Fritz-Ulrich Hahne, Gabor Nardai, Gabriel Chiriac, Gabriel Petre Gorexki, Gabriele Racanelli, Gabriele Wolz, Gabriella Bottari, Gabriella Parisi, Garri Slobodianiuk, Genoveva Cadar, Georg Fritz, Georg Kreuzer, George Notas, George Sakka, Georgi Popivanov, Gerd Klinkmann, Ida Giacinto, Michele Giannini, Gideon Latten, Giovanna Maddalena, Gisa Andresen, Giuseppe Brisinda, Giuseppe Lauria, Goffredo Angioni, Göran Stenlund, Grzegorz Płoński, Günther Oprea, H.A.H. Kaasjager, H.R. Bouma, Haitham Mutlak, Hakan Ekvall, Hana Chmelíčková, Hannes Gabriel, Nolan Hassold, Hauke Rensing, Heike Schlegel-Höfner, Heiko Groth, Heiner Ruschulte, Heinrich V. Groesdonk, Hendrik Rüddel, Henning Jansen, Henrik Rueffert, Herbert Glenny Baquerizo Vargas, Iacopo Cappellini, Ibolya Toth, Ilka Schanz, Illes Hajnal-Gabriela, Ioana Grigoras, Ioannis Orfanos, Irena Osojnik, Ireneusz Rzepnieswski, Iris Klaus, Iris Koper, Iulia Bagiu, Iulia-Roxana Morlova, Ivan Herold, Ivana Pavlić, Ivo Schindler, Izzaddin Mohd, J. Kraßler, J. Miquel Morales, Jacek Prokopowicz, Jacob Packmohr, Jacobien Hoogerwerf, Jadranka Pavicic Saric, Jakub Bilski, Jan Beneš, Jan Breuls, Jan Castan, Jan Frič, Jan Mersmann, Jan P. Roesner, Jan Seemann, Jan Zatloukal, Jan-Hinrich Baumert, Jan-Jakob Meyer, Jana Fastnacht-Böttcher, Jana Protzel, Janos Szederjesi, Janusz Gruszczyk, Janusz Michalak, Jaroslav Houser, Jarosław Janc, Jarosław Luboński, Javier Hermida Yañez, Javier Pilar, Jean-Louis Vincent, Jean-Philippe Lanoix, Jelmer Alsma, Jens Christian Kubitz, Jens Döffert, Jens Eriksson, Jens Heidrich, Jens Soukup, Jeppe SA. Neverlsen, Jeremie Pasquier, Jesper Svefors, Jesús Emilio Barrueco Francioni, Jiří Dvorský, Jiří Klimeš, Jiří Pařízek, Jiri Zurek, Joanna Bartczak, Joanna Zorska, João Gamito Lopes, Joao Miguel Ribeiro, Johan Berkius, Johan Lindström, Johann Knotzer, Johanna Hästbacka, Johannes Rhein, Jolanta Wierzchowska, Jona Weißkirchen, Jonas Sunden-Cullberg, Jonas Tverring, Jörg Engel, Jörg Kappert, Jörg Rehlinghaus, José Artur Paiva, José Pedro Cidade, Josep Trenado, Juan Carlos Yebenes, Juan Fernando Gerber, Juan Jose Diaz, Juan Pablo Horcajada, Judit Kovacs, Julika Schön, Karel Maelegheer, Karina Stefańska-Wronka, Karl Reiter, Katarzyna Baścik, Katarzyna Skawińska, Katia Donadello, Katja Masjosthusmann, Kay-Lars Müller-Forte, Kelly Straka, Ken Dewitte, Kerstin Achmus-Stenz, Kim Jie, Kitty Slieker, Klaus Kogelmann, Knut Mauermann, Knut Müller, Koronakis Nikolaos, Kresimir Oremus, Kuido Nõmm, Lars Ljungström, Lars Witt, Laura Popa, Laura Ryan, Lavinia Ionescu, Lenneke van Lelyveld-Haas, Leo Licari, Liana Valeanu, Libor Zahradníček, Line Samuelsson, Lisa Mellhammar, Liudmila Hasak, Livia Vladoiu, Lorena Forcelledo Espina, Ludger Kämmerling, Luigi Bonavina, Luis Ensenat, Lukas Barnas, Lukas Lörinc, Łukasz Olendrzyński, Łukasz Rak, Lupe Coronel, Lutz Pfeiffer, M. Gryskova, M. Schüler, Maarit Hult, Maciej Gawor, Magdalena Miulescu, Magdalena Wujtewicz, Magnus von Seth, Maja Karaman Ilic, Malgorzata Mikaszewska-Sokoleiwcz, Malte Morisse, Matthias Meyer-Barner, Manelli Filippo, Manfred Hoefler, Manfred Nuscheler, Corina-Cecilia, Manole-Palivan, Mary Nicoleta Lupu, Marc Beckers, Marc Bota, Marco Barchetti, Marco Ingrosso, Marco Ripa, Maria Andersson, Maria De Marco, Maria Fortofoiu, Maria Galiana Ivars, María José Pérez Pedrero, María Juliana Hernández Benito, Maria Slocker Barrio, Maria Zach, Mariana Anghele, Mario Guarino, Mariola Maciaszek, Mariola Tałałaj, Marisa Ann Capaldi, Marius Papurica, Mark Neufang, Markus Bressler, Markus Eller, Markus Kestler, Markus Kredel, Markus Rudnick, Markus Teipel, Marta Valcarcel Rodriguez, Martijn van Tellingen, Martin Beiderlinden, Martin Bier, Martin Cermak, Martin Ebert, Martin Helan, Martin Kahl, Martin Metelka, Martin Reichert, Martin Schütz, Martin Spångfors, Martin Wagner, Martina Čehulić, Martina Pavletić, Marton Gyenge, Mary Bedding, Marzouk Mehdi, Maša Biberič, Mateusz Miszczyszyn, Mathias Fischer, Mathias Sprenger, Matija Belavić, Matija Jurjević, Matjaž Groznik, Matthias Gründling, Matthias Huck, Matthias Lutze, Matthias Menzel, Matthias Schmauß, Matthias Weiß, Maurizio Mazzoni, Max Müller, Jochen Souquet, Maximilian Ragaller, Mercedes Ibarz Villamayor, Michael Booke, Michael Buerke, Michael Dalager-Pedersen, Michael Dolch, Michael Fries, Michael Habenicht, Michael Kremer, Michael Meisner, Michael Meyer, Michael Pogatschnigg, Michael Schütz, Michael Stofkoper, Michal Holub, Michal Pelíšek, Michal Sitina, Michał Śpiewak, Michał Ziółkowski, Michelle Behan, Miguel Castelo-Branco Sousa, Mihaela Blaj, Mihaela Conci, Mihaela Lupse, Mika Valtonen, Mikael Moriconi, Dagmar Minaříková, Miroslav Zajicek, Mirza Aun Muhammad Baig, Mitre Ioan Remus, Modesto Martinez Pillado, Mohamed Hasaballah, Monique van der Lugt, Moritz Unglaube, Natacha Mrozek, Nádia Guimarães, Nahema Issa, Nathalie Layios, Naya Bellaubí Pallarés, Nelson Barros, Nieves Carbonell Monleón, Nicolas Belaube, Nicolas Mongardon, Nicolas Serck, Nikolaas Verbeke, Nikolas Lambiris, Nikoletta Rovina, Nils Haake, Nina Polze, Nina Sulen, Noelia María Muñoz Guillen, Nora Bruns, Noud Buenen, Ojan Assadian, Ole Broch, Olena Oliwa, Olga Klementová, Oliver Beck, Oliver Habler, Oliver Hensel, Oliver Kunitz, Oliver Schmid, Olof Blivik, Ondrej Zika, Onnen Mörer, Orestis Ioannidis, Oskar Ljungquist, Pablo Vidal Cortés, Paola Rodari, Papa Raffaella, Pär Lindgren, Pascal Stammet, Pasquale Minchella, Patricia Patricio, Patrik Gille-Johnson, Patrizia Petricci, Paula Pestana, Paula Vera, Pavel Dostal, Pavel Malina, Pavel Sedlák, Pedro Castro Rebollo, Pedro Rascado Sedes, Peter Brand, Peter Neumann, Petr Hubacek, Petr Stepanek, Petr Uhlig, Petra Wegermann, Philipp Tsafoulis, Pierre Fillatre, Pierre Gregoire Guinot, Pierre Tattevin, Pio Faust, Piotr Czempik, Piotr Smuszkiewicz, Piotr Staniewski, Plinio Calligaro, Polychronis Tassioudis, Přemysl Dörr, Przemysław Michalski, R. Klimaschewski, R. Schnabel, Radek Cihlář, Rafael Leon Lopez, Rafał Gałuszka, Rafał Wójtowicz, Rainer Haas, Ralf Walper, Ralph Budnik, Ralph Sander, Raphael Gukasjan, Reiner Sitzler, Reinoud Cartuyvels, Renata Szmigielska, Renate Koch, René Verbeek, Ricard Ferrer, Ricardo Castelbón, Rob Wilting, Robert Bocek, Robert Janda, Roberta Moretti, Roelie Wösten-van Asperen, Romain Jouffroy, Romaric Larcher, Ronald Seidel, Rosario Cristoforo Puleo, Rouven-Alexander von Holten, Roxana Ungurean, Rüdiger Albrecht, Rüdiger Lott, Rui Terras, Rute Fernandes Alves, Ruth Müller, Ryszard Tokarczuk, Salla Kumpulainen, Sami Mäenpää, Sandra Müller, Sandra O. Donovan, Sanna Pohjanpaju, Sara Cajander, Sascha Ussat, Saviano Angela, Sebastian Brandt, Sebastian Casu, Sebastian Hafner, Sebastian Hammerich, Sebastian Rehberg, Sebastian Tranca, Selena Knoth, Shahram Mashayekhi, Shona Fitzgerald, Silvana Pilia, Silvia Carbajo Azabal, Silvia De Rosa, Simo-Pekka Koivisto, Simon Oelsner, Simon Schäfer, Simona Barnini, Simona Viola, Simu Tudor, Sinead O. Neill, Stanisław Iwańczak, Söhnke Boye, Sonja Krofak, Spadaro Savino, Staffan Tevell, Stefan Angermair, Stefan Bergt, Stefan Markoff, Stefan Ostmeier, Stefan Otto, Stefan P. Wirtz, Stefan Rußwurm, Stefan Schroeder, Stefan Staar, Stefanie Vandervelden, Stefano Trapassi, Steffen Volkert, Stepani Bendel, Stephan Baumann, Stephan Blache, Stephanie Koster, Stephanie Mifsud, Sulamita Carvalho Brugger, Sunil Jagoda, Sven Ackermann, Sybille Glöckl, T. Dormans, Tajana Milanovic Robina, Talvikki Koskue, Tatiana Ciomartan, Teresa Tomasa, Theodoros Aslanidis, Thessa VerNeverst, Thomas Eckermann, Thomas Grau, Thomas Hahn, Thomas Kiss, Thomas Libuda, Thomas Melzer, Thomas Rösel, Thomas Uhlig, Thomas Volk, Thomas Weidermann, Thorsten Keil, Tim Bruckmoser, Tim Piepho, Timmermans Mark, Tina Frolo, Tina Tomić Mahečić, Toamas Gabrhelik, Tobias Gropp, Tobias Klöpper, Tobias Lahmer, Tobias Leipold, Tomáš Málek, Tomáš Tajč, Tomas Urbina, Tomas Vymazal, Tomasz Musiuk, Tomasz Siegel, Tomasz Skalec, Torsten Fricke, Udo Gottschaldt, Ulf Linstedt, Ulrich Blumenthal, Ulrich Katt, Ulrich Plog, Ulrich Ronellenfitsch, Ursula George, Ute Brüggemann, Uwe Treue, Václava Adámková, Valeria Caramello, Vasileios Kaldis, Vasileios Nolas, Vasilios Papaioannou, Verena Plötz, Vesna Degoricija, Viktor Švigelj, Ville Jalkanen, Vincenzo Francesco Tripodi, Vladimír Zemánek, Volker Ostermoor, W. Alexander Osthaus, W. Joost Wiersinga, W.B. Huiszoon, Wahid Altaf, Waldemar Sobolewski, Walravens Stig, Walter Stadlmeyer, Wiktoria Mizak, Witold Wasiak, Wojciech Dabrowski, Wolfgang Heinke, Wolfgang Zink, Xavier Muschart, Yuriy Bunin, Yvo Boué, Yvonne Young, Zdenek Kos, Zdenko Povšić-Čevra, Zrinka Safaric Oremus, Zuzanna Górska

**Affiliations:** a Department of Anaesthesiology, Intensive Care Medicine, Emergency Medicine and Pain Medicine, University Medicine Greifswald, Greifswald, Germany; b 4th Department of Internal Medicine, National and Kapodistrian University of Athens, Medical School, Athens, Greece; c Service de Réanimation, Hôpital Raymond Poincaré, Assistance Publique—Hôpitaux de Paris, Université Versailles Saint-Quentin, Garches, Versailles, France; d Critical Care Center, Parc Taulí Hospital Universitari, CIBERes, Institut d'Investigació i Innovació Parc Taulí (I3PT-CERCA), Autonomous University of Barcelona, Sabadell, Spain; e Department of Internal Medicine, Hacettepe University, Ankara, Türkiye; f Paediatric Intensive Care Unit, Department of Emergency and General Pediatrics, Bambino Gesù Children's Hospital, IRCCS, Rome, Italy; g Department of Internal Medicine, Clinical Pharmacy and Pharmacology, University Medical Center Groningen, University of Groningen, Groningen, Netherlands; h Dept. of Anaesthesia and Intensive Care Medicine, Charles University in Prague, 3rd Faculty of Medicine, Prague, Czech Republic; i Constantine the Philospher University in Nitra, Faculty of Social Sciences and Health Care, Slovak Republic; j Department of Anaesthesiology, Resuscitation and Intensive Care, Zagreb University Hospital Centre, Zagreb, Croatia; k Emergency Department, Antwerp University Hospital, Antwerp, Belgium; l Department of Anaesthesiology and Intensive Care, “Prof Dr CC Iliescu” Emergency Institute for Cardiovascular Diseases, Bucharest, Romania; m University of Medicine and Pharmacy Carol Davila, Bucharest, Romania; n Department of Anesthesia and Intensive Care, Tampere University Hospital, Wellbeing Services County of Pirkanmaa and Tampere University, Tampere, Finland; o Tours University, School of Medicine and Tours University Hospital, CHU Tours, Emergency Medicine Department, 37044, Tours, France; p Department of Emergency Medicine, Odense University Hospital, Odense, Denmark; q North-West State Medical University named after I.I. Mechnikov, Federal Research and Clinical Center of Intensive Care Medicine and Rehabilitology, Saint Petersburg, Russian Federation; r Division of Infection Medicine, Faculty of Medicine, Department of Clinical Sciences Lund, Lund University, Lund, Sweden; s Department of Anaesthesiology and Intensive Care Medicine, Faculty of Medicine, University Hospital Ostrava and University of Ostrava, Ostrava-Poruba, Czech Republic; t First Department of Anaesthesiology and Intensive Therapy, Medical University of Lublin, Lublin, Poland; u Medical Data Management, Medaman, Geel, Belgium; v Anestesia e Rianimazione dei Trapianti Dipartimento Chirurgico Polispecialistico ASST Grande Ospedale Metropolitano Niguarda Milano, Milan, Italy; w Department of Critical Care, King's College London, Guy's & St Thomas' Hospital, London, United Kingdom; x Division of Internal Medicine, University Hospital Basel and Department of Biomedicine and Department of Clinical Research, University of Basel, Basel, Switzerland; y Intensive Care Medicine Service, Centro Hospitalar Universitário São João, Porto, Portugal; z Department Infectious Diseases, Italian National Institute of Health, Rome, Italy; aa Clinical Department of Anesthesiology and Intensive Therapy, Wroclaw Medical University, Wroclaw, Poland; ab Institute of Bioinformatics, University Medicine Greifswald, Greifswald, Germany; ac Clinic for Anesthesiology, Reanimation and Intensive Care, University Clinical Center Niš, Niš, Serbia; ad Section for Medical Microbiology, Department of Laboratory Medicine Nord-Trøndelag Hospital Trust, Levanger, Norway; ae Department of Medical Microbiology, St Olavs Hospital, Trondheim University Hospital, Trondheim, Norway; af Charité Universitätsmedizin Berlin, Department of Anesthesiology and Operative Intensive Care Medicine, Berlin, Germany; ag Intensive Care Department, Vall d’Hebron Hospital Universitari, Barcelona, Spain; ah Friedrich Loeffler-Institute of Medical Microbiology, University Medicine Greifswald, Greifswald, Germany; ai Institute of Medical Microbiology, University Hospital Münster, Münster, Germany; aj SODIR Research Group, Vall d’Hebron Institut de Recerca (VHIR), Barcelona, Spain; ak Department of Medicine, Universitat Autònoma de Barcelona Vall d’Hebron Barcelona Hospital Campus, Barcelona, Spain

**Keywords:** Sepsis, Blood cultures, Diagnostics, Infrastructure, Turn-around time, Opening hours, Rapid testing, Single-site sampling, Multiple-site sampling, Number of blood cultures sets, Clinical practice, Pre-analytical practice, Blood culture analysis

## Abstract

**Background:**

Blood cultures (BCs) are key diagnostic elements for sepsis patients. Accurate preanalytical procedures are substantial, and results should be available as soon as possible to guide adequate antimicrobial treatment. This study aimed to evaluate BC collection practices and diagnostic capacity across European hospitals.

**Methods:**

This cross-sectional survey investigated BC diagnostics in acute care hospitals across 37 European countries in the years 2021 and 2022. Analyses included BC guidelines, collection sites, number of BC sets in emergency departments (EDs), wards, and intensive care units (ICUs). We also examined transfer after collection, the use of on-site vs. external laboratories, opening hours, rapid testing capacity, and turn-around times of BCs processed in microbiology laboratories with different infrastructures.

**Findings:**

Responses were collected from 907 hospitals in Europe. BC guidelines were available in 84·4% (741/878) of the hospitals. BCs were preferably collected by multiple-site sampling in EDs (62·7%, 461/735), in wards (64·0%, 513/802) and ICUs (68·5%, 518/756). One BC set was preferred in EDs in 38·4% (270/704), in wards in 40·5% (314/775), and ICUs in 34·9% (261/748). Two BC sets were preferred in EDs in 31·0% (218/704), in wards in 28·1% (218/775), and ICUs in 39·2% (293/748). 48·0% (402/838) of hospitals used on-site and 52·0% (436/838) external microbiology laboratories. Around-the-clock microbiological services were available in 10⋅0% (91/907), and rapid pathogen identification in 43·7% (396/907) of hospitals. Infrastructure with around-the-clock microbiological service and rapid testing was available in 7·4% (62/840) of hospitals, and probability of a final microbiological result within two days was highest in these hospitals compared to hospitals with limited microbiology service (for BC collected on wards: 19·6% vs. 52·7%, Odds Ratio 4·59 [95% CI 2·50–7·79], p < 0·0001).

**Interpretation:**

Despite the availability of BC guidelines in many hospitals, current recommendations for BC collection were often neglected. Rapid testing capacity was limited in most microbiological laboratories, and around-the-clock service for BCs was very rare. As delay in results may have a detrimental impact on patient outcomes, strategies to improve these processes are urgently needed.

**Funding:**

The European Sepsis Alliance and a grant by Becton and Dickinson.

**Research in context**

**Evidence before this study**

Blood culture collection is a key element in sepsis diagnostics, as it supports the identification of the pathogen causing the infection. Appropriate preanalytical practices and rapid processing of blood cultures are essential to provide clinicians with valid microbiological results as promptly as possible. We conducted a PubMed search for studies published between January 2000, and September 2025 that reported on preanalytical practices of blood culture collection in patients with sepsis and/or on the infrastructure and capacity of microbiological laboratories processing blood cultures. We used the search terms “(“blood culture”) AND (“practice” OR “infrastructure“ OR “capacity“ OR “microbiological diagnostics“), and limited the search to multi-centre studies describing practices and infrastructure at the level of countries. Up to now, there was one study in 2013 that investigated 79 microbiological laboratories and 59 ICUs across four European countries. This study revealed considerable differences in the quality of blood culture testing. In particular, time to incubation was a considerable problem, largely due to the increasing number of remote laboratories. A 2017 study from Germany involving 706 medical doctors and final-year medical students suggests that there are substantial deficits in blood culture ordering and in the application of guidelines for blood culture practice. The largest study, published in 2019, collected data from 209 laboratories across 25 European countries on microbiological diagnostics of bloodstream infections at the laboratory level. This study demonstrated that laboratories have begun to use novel methods for rapid identification and antimicrobial susceptibility testing. However, this improvement was hampered by limited operating hours, meaning that current practices of blood culture diagnostics in Europe only partially meet the requirements for optimal management of bloodstream infections. A 2022 study from Nordic countries investigated blood culture diagnostics in 49 clinical microbiology laboratories in Denmark, Finland, Iceland, Norway and Sweden. The study reported limited opening hours and restricted availability of clinical microbiologists. Overall, there is little comparative information on the practice of blood culture diagnostics in Europe, taking into account the clinical aspects, the processes and the available laboratory infrastructure.

**Added value of this study**

Compared to previous studies, we extended the analyses to include both blood culture processing in microbiology laboratories and preanalytical practices of blood culture collection in hospitals, differentiating between emergency departments, wards, and intensive care units. We increased the number of countries studied and included a substantial sample of European hospitals of all sizes and types. This study provided a comprehensive analysis of blood culture diagnostics including preanalytical aspects, transportation, as well as infrastructure of microbiology diagnostics and its availability. The differences in laboratory infrastructure observed were contextualized with the reported turn-around times of blood cultures. This approach allowed us to demonstrate the impact of microbiology laboratories providing 24/7 service and using rapid testing on turn-around times, in contrast to laboratories with limited capacity.

**Implications of all the available evidence**

In this study, involving a large sample of European hospitals of all categories, preanalytical practices of blood culture collection were often not in accordance with current recommendations. Particularly the recommended number of blood culture sets was frequently not followed. Shipping to laboratories was often delayed, and most laboratories had limited opening hours. The results of our study, along with previous research on these aspects, provide convincing evidence that there is substantial room for improvement at preanalytical stages of blood culture diagnostics. Additionally, there is potential for enhancing the capacity of microbiology laboratories, including extending opening hours and implementing rapid testing. These findings are important because delays in time to result may have a detrimental impact on patient outcomes. Urgent measures at national and international levels are needed to ensure timely and effective care for patients with sepsis.

## Introduction

Blood cultures (BC) are the gold standard for detecting bloodstream infections, especially in patients with sepsis. They are important for identifying the causative pathogens and support the correct choice of antimicrobials.^1^ Although broad-spectrum antibiotics are in most cases part of the initial treatment of sepsis, in a substantial number of cases, antibiotic prescriptions have gaps in coverage or encounter pathogens resistant to the administered agents, resulting in inadequate treatment.^2^^,^^3^ The BC findings inform management, guide clinicians to tailor antimicrobial therapy for optimal efficacy, and allow de-escalation of antimicrobials to minimize collateral damage.^4^^,^^5^ There are several practice guidelines about how to collect a BC,^6^, ^7^, ^8^, ^9^, ^10^, ^11^ but many aspects related to the process of taking BCs are subject of intensive research and scientific debate. This includes, for example, the collection site, the number of punctures (single-site vs. multiple-site sampling), the optimal number of BC sets and blood volume.^1^^,^^12^, ^13^, ^14^, ^15^, ^16^, ^17^, ^18^, ^19^, ^20^, ^21^, ^22^, ^23^, ^24^, ^25^ BC analysis is a complex process, involving multiple steps that require considerable time and expertise. There are only limited data regarding BC sampling strategies, microbiological handling of BCs, laboratory operating hours, and the use of rapid testing for BCs in European countries.^26^, ^27^, ^28^, ^29^ Overall, there is little comparative information on the practice of blood culture diagnostics in Europe, taking into account the clinical aspects, the processes and the available laboratory infrastructure.

However, such organizational and infrastructural aspects are essential for the optimal management of sepsis patients.^1^^,^^26^^,^^30^

In this study, we aimed to evaluate practices in BC collection and diagnostic capacity across European hospitals and examine their relationship with diagnostic turnaround time.

## Methods

### Design and population

The European Sepsis Care Survey (ESCS) was a cross-sectional survey project investigating sepsis care in acute care hospitals in a convenience sample. A self-reported 94-item questionnaire (see Supplemental Material), harmonized by a multiprofessional steering committee led by the European Sepsis Alliance and peer-reviewed by the scientific committees of four European professional scientific societies (see Supplemental Material) was distributed to health-care professionals working in acute care hospitals in 69 countries. Participants registered online and completed the survey via a web-based platform (LamaPoll Platin Edition, Germany) using a hospital-specific, unique access link. They were encouraged to obtain the necessary details from other services or departments within the hospital. Each hospital was permitted to submit only one entry, including sections for the emergency department, wards, and intensive care units. The data were collected in 2021 and 2022, and were handled confidentially. The questionnaire contained questions about sepsis screening and sepsis management, as well as BC collection practices and the diagnostic capacity of microbiology laboratories. The general part of the ESCS was published previously.^31^ Here, we report the analysis of ESCS data regarding BC collection practices and microbiological diagnostics. The study protocol received ethical approval from the ethics committee of University Medicine Greifswald, Germany (BB 124/21), where data were stored and processed. Written consent from the participants was not required. The ESCS complied with the EQUATOR network reporting guidelines CHERRIES^32^ and CROSS^33^ and was registered at ClinicalTrials.gov (Identifier: NCT05059808). For this analysis we used data from acute care hospitals located in 37 countries in Europe, including Albania, Austria, Belarus, Belgium, Bulgaria, Croatia, Cyprus, Czechia, Denmark, Estonia, Finland, France, Germany, Greece, Hungary, Iceland, Ireland, Italy, Luxembourg, Malta, Netherlands, North Macedonia, Norway, Poland, Portugal, Republic of Moldova, Romania, Russian Federation, Serbia, Slovakia, Slovenia, Spain, Sweden, Switzerland, Türkiye, Ukraine and United Kingdom. Data from countries outside the European continent were excluded due to fundamental differences in procedures used in European and non-European countries.

### Variables of interest

The following aspects were analysed: availability of a hospital protocol or guideline for the collection of BCs, BC collecting staff, BC sampling sites including single-site vs. multiple-site sampling, the number of BC sets per collection and surveillance of the number of BCs. We further investigated the processing of BC after collection, the availability of on-site or external microbiology, transfer priority to the microbiology, and the availability of around-the-clock microbiological service. Around-the-clock service was defined as including BC incubation, pathogen identification, antimicrobial susceptibility testing (AST) and result reporting. We also assessed the availability of rapid testing, i.e., rapid pathogen identification by PCR, direct MALDI-TOF MS, or MALDI-TOF MS after short incubation, and rapid AST directly from positive blood cultures, as well as BC tracking and reporting systems. Turn-around-times (TAT) to preliminary results (e.g., Gram stain) and TAT to final microbiological result (including pathogen identification and AST) reported by the participants were analysed.

### Statistical analyses

Analyses were performed by using Excel Version 16, RStudio and Datawrapper. Due to the lack of standardised information on the total number of hospitals in the individual countries and therefore the impossibility of making a statement on the response rate, representativity per country was calculated based on number of beds in the included hospitals divided by total bed capacity per country according to Organisation for Economic Co-operation and Development (OECD) and European Statistics Office (Eurostat) data (Supplemental Table S2). Only definitive answers (yes/no) were used to calculated proportions. Responses of the category “I don't know” were not considered since they could shift the results in either direction. Since not all questions where mandatory, the number of participants included in each analysis varied. Results were presented as numbers and proportions. To avoid biased representation from countries with only a few participating hospitals, we presented country-specific results only if a country contributed at least 10% of its total hospital bed capacity, or at least 10 hospitals.

In a second step, we put the available diagnostic infrastructure and capacities in context with the BC TAT. The TAT of different settings (with and without around-the-clock BC processing; with and without rapid testing) were compared. Odds ratios with 95% confidence intervals were calculated for the probability of having a microbiological result within two days. Hospitals without 24/7 microbiology service and without rapid testing were set as reference. The Chi-squared test was used to compare contingency tables. p-values below an alpha level of 0·05 were considered statistically significant. Due to the multiple comparisons, a Bonferroni correction was performed to adjust all p-values.

### Role of the funding source

The study was supported by a grant from Becton Dickinson SA for technical realisation of the study. Becton Dickinson SA was not involved in the study design or recruitment or in writing, reviewing, or submission of the manuscript. This article represents the views of the named authors only.

## Results

The original ESCS included 1023 hospitals worldwide. The present study analysed only European data including 907 hospitals from 37 European countries (713 hospitals from 26 countries within the European Union and 194 hospitals from 11 non-EU countries). Participants were hospital directors (6·0%, 54/907), head or deputy head of departments (44·4%, 403/907), consultants (38·6%, 350/907) and other (i.e., doctors, residents, nurses) (11·0%, 100/907). Details about the positions and specialties are provided in the Supplemental Material (Table S1).

The study included 312 (34·4%) university and 595 (65·6%) non-university hospitals. We included 260 hospitals with less than 250 beds, 268 hospitals with 251–500 beds, 140 hospitals with 501–750 beds, and 239 hospitals with more than 750 beds (Supplemental Table S3). Based on data from the OECD on hospital bed capacity, the study covered 13·9% of all acute care beds in the included countries. In the European Union, the sample covered 23·1% (388,574 out of 1,681,334) of all acute care beds in the 26 European Union countries and 6·2% of the capacity in the non-European Union countries (Supplemental Figure S1 and Table S2).

### Blood culture guidelines

**Fig. 1:**
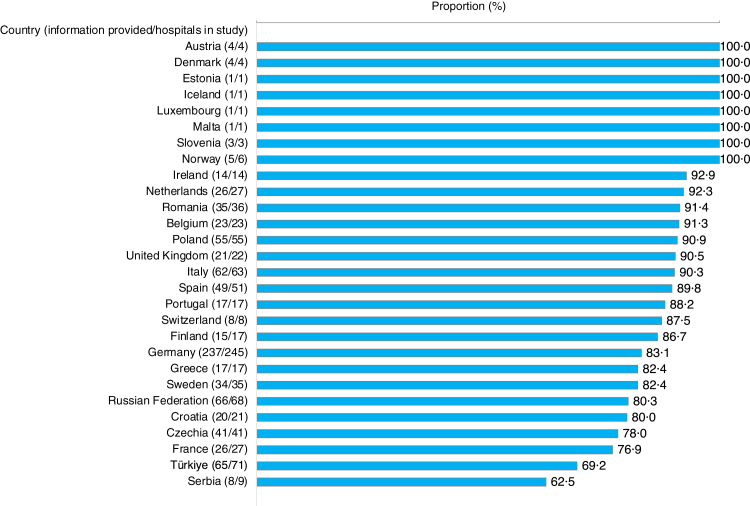
**Availability of blood culture guidelines or protocols by country**. The figure shows only countries that contributed at least 10% of the country's hospital bed capacity, or at least 10 hospitals of the country. Due to this threshold, data from Bulgaria, Cyprus, Hungary, Slovakia, Belarus, Ukraine, Republic of Moldova, North Macedonia and Albania are not presented in the figure. Proportions were calculated on definitive answers (yes/no). The numbers in brackets present the “number of hospitals that provided information/total number of hospitals in the study”.

Information about the availability of a guideline or protocol for the collection of BCs was reported from 878 hospitals. 84·4% (741/878) of the hospitals reported to have a BC guideline or protocol. In individual countries, implementation of such guiding documents varied between 55·6% and 100% (Fig. 1). BC guidelines were available in 93·3% (42/45) of private hospitals, followed by university hospitals (87·5%, 265/303), and general or community hospitals (81·9%, 434/530) (p = 0·61). The hospital bed size had no significant influence on the availability of guidelines (p > 0·99).

### Blood culture collection practices

**Fig. 2:**
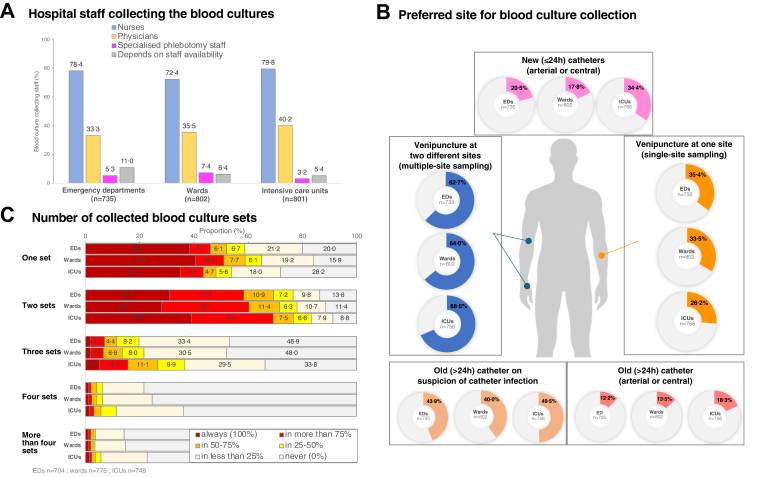
**Blood culture collection practice in case of sepsis**. (A) The majority of BCs were collected by nurses. (B) Preferred site for BC collection was a multiple-site approach. In case of suspected catheter infection, a collection from more than 24 h old catheters was also frequently reported. Alternative collection sites for BCs are presented in the Supplemental Table S4. (C) In emergency departments (EDs), on wards, and intensive care units (ICUs), one or two blood culture sets were collected in most cases. More information about the number of BCs sets is presented in the Supplemental Table S5.

BCs were predominantly collected by nurses (emergency departments (EDs) 78·4% (576/735), wards 72·4% (581/802), intensive care units (ICUs) 79·8% (639/801), respectively), with a higher proportion of BC collecting by nurses in EDs and on ICUs (p = 0·029). Physicians collected BCs in EDs in 33·3% (245/735), on wards in 35·5% (285/802), and on ICUs in 40·2% (322/801), respectively) (p = 0·41). Specialized phlebotomy teams were rarely utilized (Fig. 2A).

Direct venipuncture at two different sites (multiple-site sampling) was the preferred method of taking blood cultures in EDs (62·7%, 461/735), on wards (64·0%, 513/802), and ICUs (68·5%, 518/756). Venipuncture at one site (single-site sampling) was the preferred technique in 35·4% (260/735) of the EDs, 33·5% (269/802) of the wards and 26·2% (198/756) of the ICUs. In case of suspected catheter infection, BCs were taken from more than 24 h old catheters in 43·9% (323/735) of the EDs, in 40·0% (321/802) of the wards, and in 49·5% (374/756) of the ICUs. Other preferred sites for BC collection are presented on Fig. 2B and alternative sites in the Supplemental Table S4. Responders indicated that they preferred a specific number of BC sets to be taken but acknowledged that this was not always achieved (Fig. 2C). Collection of only one BC set was reported from wards in 40·5% (314/775), from EDs in 38·4% (270/704), and ICUs in 34·4% (261/748) (p > 0·99). Collection of always two BC sets was reported from EDs in 31·0% (218/704), from wards in 28·1% (218/775), and from ICUs in 39·2% (293/748) (p = 0·0002). Sampling of three and more BC sets was less frequently reported (Fig. 2C and Supplemental Table S5).

### Blood culture transfer, tracking, result reporting and quality indicators

**Fig. 3:**
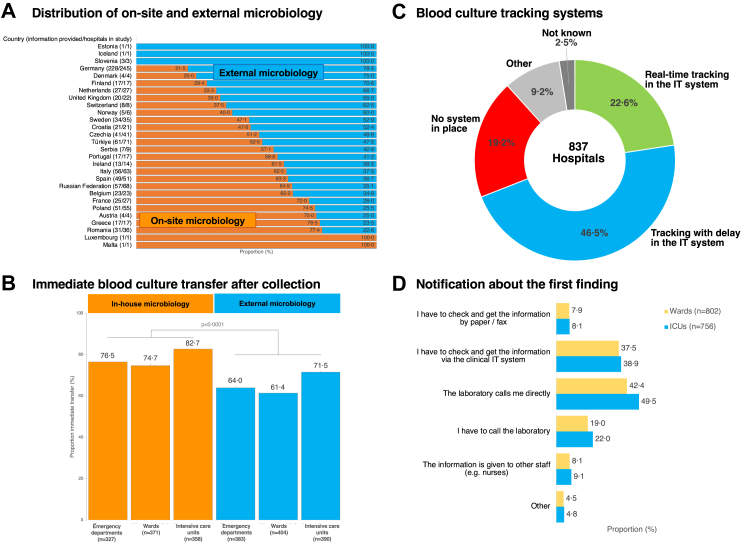
**Blood culture transfer, tracking and reporting**. (A) Distribution of on-site and external microbiology laboratories providing service for hospitals in different countries. (B) Transfer priority of blood cultures to the microbiology after collection. Displayed are the proportions with immediate transfer. In other cases, blood cultures were transported with delay. For more information see Supplemental Table S5. (C) Availability of blood culture tracking systems to view the status of blood cultures. (D) Communication of information regarding the first microbiological finding to a clinician. Figure (A) shows countries that contributed at least 10% of the country's hospital bed capacity, or at least 10 hospitals of the country. Due to this threshold, data from Bulgaria, Cyprus, Hungary, Slovakia, Belarus, Ukraine, Republic of Moldova, North Macedonia and Albania are not presented in the figure. Proportions were calculated on definitive answers (yes/no). The numbers in brackets present the “number of hospitals that provided information/total number of hospitals in the study”.

Among the hospitals, 838 provided information about the location of their microbiology laboratory, 48·0% (402/838) used on-site microbiology laboratory and 52·0% (436/838) used external microbiological services to process BCs (p > 0·99). The ratio of on-site and external laboratories in individual countries and hospital sizes varied and is shown in Fig. 3A and Supplemental Material Table S7. Responders reported that BCs were sent to the microbiology laboratory immediately after collection from the EDs (69·7%, 495/710), from the wards (67·7%, 525/775), and from the ICUs (76·9%, 575/748) (p = 0·14). In smaller proportions, BCs were sent at set times throughout the day, were sent irrespective of collection time, or were stored in the department where they had been taken and collected by personnel from the laboratory depending on availability (Supplemental Table S6). Immediate transfer of BCs to the laboratory was reported more frequently in hospitals with on-site microbiology compared to hospitals with external microbiology (EDs 76·5% (250/327) vs. 64·0% (245/383), p = 0·008); wards (74·7% (277/371) vs. 61·4% (248/404), p = 0·002); and ICUs (82·7% (296/358) vs. 71·5% (279/390), p = 0·008) (Fig. 3B).

The actual state of the BC processing could be tracked in 22·6% (189/837) of hospitals in the hospital information system in real-time, in 46·5% (389/837) of hospitals with delay, and 19·2% (161/837) had no system for BC tracking (Fig. 3C). The first finding (e.g., microscopy result or species identification) was reported by direct calls from the laboratory and via clinical IT systems; there were also notifications via paper and fax or by other ways (Fig. 3D).

In 47·5% (393/828) of hospitals, the number of BCs was systematically measured as quality indicator. Contamination rates within their hospitals were only reported by 15·3% (139/907) of the responders with a median contamination rate of 10% (IQR 5–18).

### Microbiological infrastructure and diagnostic capabilities

**Fig. 4:**
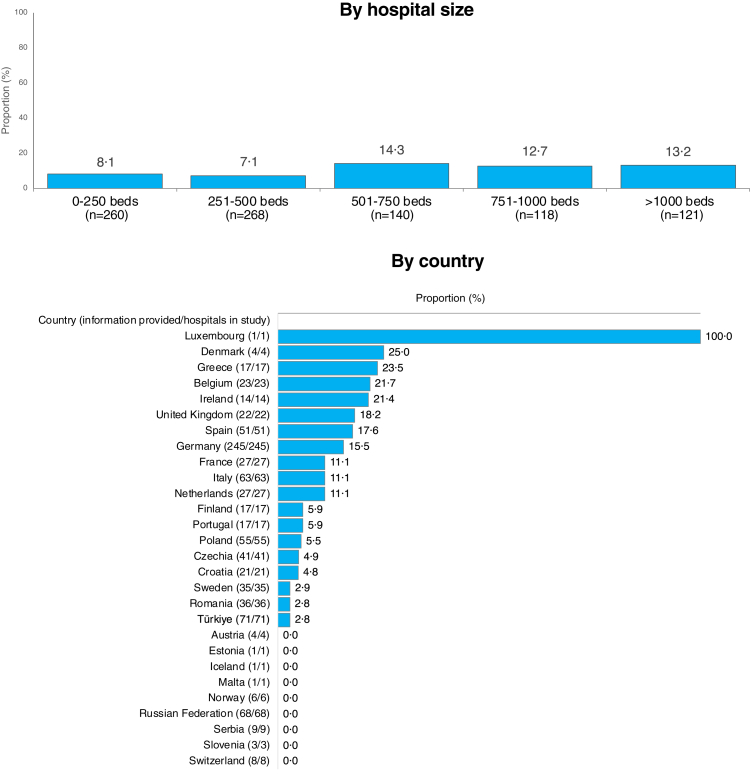
**Availability of around-the clock microbiology service**. Availability of timely unlimited (24/7) microbiological service for blood culture analysis in hospitals of different sizes and countries. Around-the-clock service had to include blood culture incubation, pathogen identification, antimicrobial susceptibility testing, and result reporting. The country figure shows only countries that contributed at least 10% of the country's hospital bed capacity, or at least 10 hospitals of the country. Due to this threshold, data from Bulgaria, Cyprus, Hungary, Slovakia, Belarus, Ukraine, Republic of Moldova, North Macedonia and Albania are not presented in the figure. Proportions were calculated on definitive answers (yes/no). The numbers in brackets present the “number of hospitals that provided information/total number of hospitals in the study”.

Around-the-clock microbiological services for blood culture incubation, pathogen identification and result reporting were available for 10·0% (91/907) of the hospitals. Larger hospitals with more than 500 beds had unlimited 24/7 service in 13·5% (51/379) compared to smaller hospitals with less than 500 beds (7·6%, 40/528) (p = 0·09) (Fig. 4). There were no significant differences in the availability of 24/7 microbiological laboratory service between university (10·3% (32/312) and non-university hospitals (9·9% (59/595) (p > 0·99), and not between on-site microbiology (9·6% (42/436) and external microbiology (10·4%, 49/471) (p > 0·99). The availability of 24/7 service in individual countries is shown in Fig. 4.

**Fig. 5:**
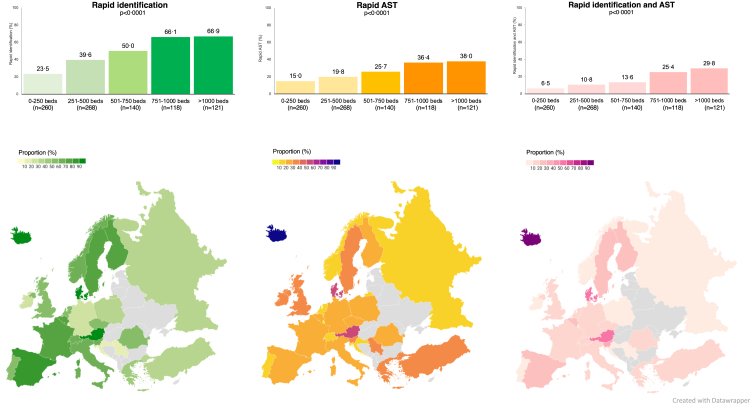
**Rapid testing capacity**. Availability of rapid pathogen identification (by PCR, direct MALDI-TOF MS, or MALDI-TOF MS after short incubation) and rapid antimicrobial susceptibility testing (AST) directly from positive blood cultures in hospitals of different size. The country maps show only countries that contributed at least 10% of the country's hospital bed capacity, or at least 10 hospitals of the country. Due to this threshold, data from Bulgaria, Cyprus, Hungary, Slovakia, Belarus, Ukraine, Republic of Moldova, North Macedonia and Albania are not shown on the maps.

**Fig. 6:**
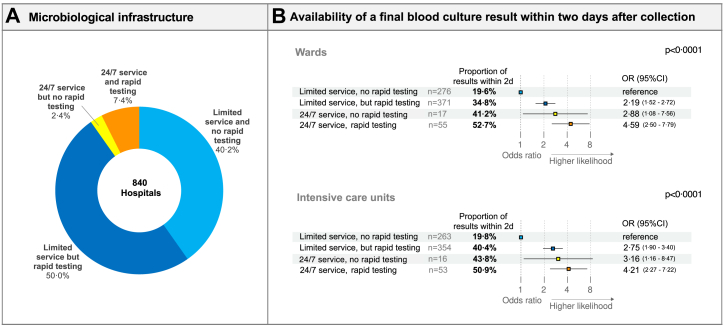
**Distribution of microbiological infrastructure and capacity for blood culture analysis and time-to-result**. (A) Distribution of microbiological infrastructure was calculated on the basis of all hospitals with complete information about around-the-clock service and rapid testing. Hospitals with partial information were not considered. (B) The first 48 h after blood culture collection were considered for the comparison of different infrastructure and capacity.

Rapid pathogen identification from positive BCs was available in 43·7% (396/907) of hospitals. AST directly from positive BCs was available in 23·9% (217/907) of hospitals. Both rapid pathogen identification and AST were available in 14·4% (131/907) of hospitals. The availability of rapid testing varied between hospitals of different sizes and across countries (Fig. 5). University hospitals compared to non-university hospitals had more rapid testing for pathogen identification (63·5%, [198/312] vs. 33·3% [198/595], p < 0·0001), AST (35·3%, [110/312] vs. 18·0% [107/595], p < 0·0001) and both, pathogen identification and AST (24·0% [75/312] vs. 9·4% [56/595], p < 0·0001). Detailed information about the overall infrastructure (opening hours and rapid testing) was provided by 840 hospitals (Fig. 6). Only 7·4% (62/840) of the hospitals had both, rapid testing and 24/7 microbiological service (Fig. 6A). On-site microbiology used more rapid testing for pathogen identification (50·5%, [238/471] vs. 36·2% [158/436], p = 0·0004), and AST (28·5% [134/471] vs. 19·0% [83/436], p = 0·02).

### Blood culture turn-around times

Participants reported how long it usually took to receive a preliminary (e.g., Gram stain microscopy) and the final BC result, including pathogen identification and AST. The majority had a final result within 3 days, on wards 69·0% (535/775) and on ICUs 72·4% (535/739) (Supplemental Figure S2).

Depending on the available infrastructure, a higher proportion of final BC results were available within two days when BCs were processed in microbiology laboratories offering around-the-clock service (24/7) and rapid testing compared to hospitals with limited service and without rapid testing (52·7% [29/55] vs. 19·6% [54/276], p < 0·0001) (Fig. 6B). The proportion of hospitals with around-the-clock microbiological service and rapid testing was only 7·4% (62/840), but the likelihood for a final microbiological BC result within two days was highest in these hospitals (wards: odd ratio 4·59 [95% CI 2·50–7·79], ICUs: odds ratio 4·21 [95% CI 2·27–7·22]). The risk of a delayed final BC result was highest in hospitals with limited microbiological services and no rapid testing, of which there were 40·2% (338/840) of hospitals (Fig. 6).

When participants were asked “*How could your microbiological diagnostic service improve?*”, 64·4% (584/907) answered “*Microbiological diagnostics of sepsis should be more rapid”*, 47·4% (430/907) felt that 24/7 microbiological service was needed, 30·5% (277/907) wanted more specific advice (e.g., on antibiotic choice, dosage, duration), 26·5% (240/907) stated that microbiological diagnostics of sepsis should be more precise, and 16·1% (146/907) were satisfied with the microbiology service.

## Discussion

This study presents a comprehensive picture of BC collection practices and microbiological capacity for BC diagnostics in Europe. In total, responses from 907 hospitals in 37 countries were analysed. With 83·0% (753/907) of participants being heads or deputy heads of departments or consultants (Supplemental Material, Table S1), the data in this study predominantly reflects input from senior professionals, supporting its validity. Until now, there has been little cross-border and comparable information on the practice of BC diagnostics. Some studies pointed to significant weaknesses that could have a relevant impact on patient safety.^26^, ^27^, ^28^, ^29^

In our study, guidelines or protocols for BC collection were available in more than 80% of hospitals, but current recommendations were often neglected and not followed. A study from Germany in 2017 already reported low adherence to the guideline recommendation of taking at least two BC sets (whereby one BC set consists of one aerobic and one anaerobic bottle).^29^^,^^34^ Also in our study there was a high proportion of EDs, wards and also ICUs, who usually collected less than two BC sets, a practice that is deviating from current recommendations,^7^ but also from recommendations that have been in place for many years.^35^^,^^36^ Interestingly, the guideline recommendation to take at least two BC sets disappeared in the latest version of the sepsis guidelines,^37^^,^^38^ which now does not clarify the needed number of BC sets.^1^^,^^13^^,^^17^^,^^39^ A potential issue of collecting only one BC set is to miss detection of bloodstream infections due to reduced blood volume and that it is challenging to differentiate contamination from true pathogen.^23^^,^^39^, ^40^, ^41^, ^42^, ^43^ Despite available evidence supporting single-site sampling, more hospitals were using multiple-site sampling, which has also been shown to increase the risk of contamination.^14^, ^15^, ^16^^,^^41^^,^^44^ Our survey revealed contamination rates (10%) higher than those recommended by the Clinical and Laboratory Standards Institute.^7^ CLSI currently recommends a contamination rate of <3% of all BCs collected. However, previous publications have reported actual overall BC contamination rates ranging from of 0·6 to 12·5%.^45^^,^^46^ Only a small proportion of respondents (15·3%) were able to provide information on contamination rates, indicating that such data are not routinely documented in many hospitals. High contamination rates are clinically significant, as they may lead to unnecessary antibiotic use, prolonged hospital stays, and increased healthcare costs, highlighting the need for systematic monitoring and targeted interventions to reduce contamination and improve patient care.^27^^,^^40^^,^^42^^,^^45^, ^46^, ^47^, ^48^, ^49^

A previous study in four European countries reported delayed BC incubation due to transportation to remote labs.^27^ Our study confirmed delayed transfer due to transport to external laboratories, but there was also significantly delayed transfer to on-site microbiological laboratories. A large survey study investigating 209 microbiological laboratories in Europe reported the use of rapid technologies in two thirds of laboratories, but only 13% had established a 24 h service to start immediate processing of positive blood cultures and only 4·7% of laboratories validated and transmitted the results of identification and AST to clinicians around-the-clock.^26^ A study in five Nordic countries in 2022 investigated 49 microbiological laboratories and confirmed limited opening hours confining the potential of satellite incubators and rapid testing.^28^ The very low number of around-the-clock microbiological service was confirmed in our study involving more countries and a larger number of hospitals. In addition, we observed an important association between the available microbiological capacity (opening hours and availability of rapid testing) and the TAT of BCs.

Hospitals with rapid testing or around-the-clock microbiological service reported significantly faster TAT compared to hospitals without these infrastructures, confirming the importance of each component. Hospitals that had both around-the-clock microbiological service for blood cultures and rapid testing reported the most accelerated TAT. The risk of a final microbiological result not being available within two days was highest in hospitals with limited microbiological opening hours and no rapid testing. This underlines the urgent need for more extensive microbiological service to provide clinicians as fast as possible with BC results to empower them to adjust antimicrobial treatments and to provide adequate antimicrobial therapyas soon as possible.^4^^,^^50^^,^^51^ This becomes more important since several studies have highlighted the increased mortality with inadequate treatment and even more importantly, the high proportion of initial inadequate antimicrobial treatment in patients with life-threatening infections.^2^^,^^52^, ^53^, ^54^, ^55^, ^56^ The time to receiving adequate antimicrobial treatment is an essential factor to successful therapy and is associated with mortality.^3^^,^^54^ It underlines the importance of extensive microbiological infrastructure, which means availability of around-the-clock microbiological service and the broad use of rapid testing for BC analysis in critically ill patients.

This study has limitations that should be taken into account when interpreting the findings. Although the data represent a broad range of European hospitals, the use of convenience sampling and voluntary participation introduces the possibility of selection bias. This study was based on a survey, not on a site audit. Therefore, responses were not validated. Not all questions were definitely answered by all respondents. If questions were answered with “I don't know” these responses were excluded from the analyses. The reality can therefore differ in either direction. As with any survey, responses may reflect more favorable or less favorable views. Information about the probability of a final microbiological BC result within two days, turn-around times, and all other data were based on the replies and estimations of the respondents and should be interpreted with appropriate caution.

### Conclusion

Although BC guidelines were available in many hospitals, current recommendations for BC collection were frequently disregarded, potentially leading to missed microbiological findings. In most hospitals, microbiology laboratories offered limited rapid testing and rarely provided 24-h BC services. Furthermore, we observed a significant association between microbiological capacity and reported BC turn-around times. These findings highlight an urgent need to improve pathogen diagnostics in patients with sepsiss.

## Contributors

CSS, KR, RF, EAI, MG contributed to the conceptualisation. CSS, DF, AL, RF, AA, SL, MO (Ostermann), MG, EGB, EAI, KR contributed to the study design and harmonised the questionnaire. DF, AL, EAI, ATA, MGS, KL JS, MG, MO (Ostermann) translated and checked the survey. CSS and national coordinators (see below) verified the data, CSS and MV had access to raw data. CSS and MV analysed the data. CSS wrote the first draft of the manuscript and produced the tables and figures.

CSS, EAI, EGB, DA, VC, MS, KZ, MO (Ostermann), MiO(Osthoff), JH, MM, MG, JM, KD, RF and RCR revised the Article.

RF, DA, AA, ATA, GB, HRB, VC, RCR, MG, KD, JH, KL, JM, MM, GM, MO (Ostermann), MiO (Osthoff), JAP, MS, JS, SL, MGS, ATL, NV and KZ contributed as national coordinators or representatives of scientific societies to survey dissemination were responsible for hospital recruitment and checked participants.

All authors contributed to data interpretation, provided substantial feedback on the article, and approved the submitted version.

CSS and EAI had the final responsibility for the decision to submit for publication.

## Data sharing statement

The data used for this analysis can be made available upon reasonable request for up to five years. For further information, please contact the corresponding author.

## Editor note

The Lancet Group takes a neutral position with respect to territorial claims in published maps and institutional affiliations.

## Declaration of interests

**Christian S. Scheer** discloses funding from European Sepsis Alliance for conducting the European Sepsis Care Survey and funding from Becton and Dickinson for technical realisation of the project. He received honoraria from BD for scientific lectures.

**Evangelos J. Giamarellos-Bourboulis** discloses grants to the Hellenic Institute for the Study of Sepsis and National and Kapodistrian University of Athens from Abbott Products Operations, bioMérieux Inc, Gentian, MSD, PHC Europe BV, Swedish Orphan Biovitrum AB (Sobi), InCyte, Novartis, Sanofi and UCB, he also discloses consulting fees and honoraria paid to the National and Kapodistrian University of Athens from Sobi, Abbott Products Operations AG, bioMérieux Inc and ThermoFisher Brahms GmbH, he is chairman of the European Sepsis Alliance (ESA).

**Djillali Annane** discloses payments to his institution from Agence Nationale de la Recherche, references ANR-18-RHUS-0004, IAHU-0004-PROMETHEUS and JTC_2021, ANR-21-PERM-0005 and honoraria from Baxter, Alexion, and Beckmann Coulter, he is member of the advisory board of Hillroom, Janssen, Pfizer, Regeneron, Sanofi, Viatris, Volition and Fab'entech.

**Antonio Artigas** discloses a study grant from Loop-Dx Ministerio De Ciencia Innovacioon Y Universidades cpp2021-008394 paid to his institution; Consulting fees from Grifols, Iilly Foundation and Aerogen for scientific advice and honoraria for lectures from Aerogen, support for attending meetings from the European Sepsis Alliance, he discloses patent planning for NANO X Sepsis, he is data safety monitoring board member of the React Trial and Modify Trial and member of the ethics and advisory board of the Immunosep Trial, he discloses receipt of equipment from biomerieux to his institution.

**Abdullah Tarik Aslan** has nothing to disclose.

**Gabriella Bottari** discloses her position as unpaid deputy chair of the infection systemic inflammation and sepsis section of the European Society of Pediatric and Neonatal Intensive Care.

**Hjalmar R. Bouma** discloses a grant from Health Holland Public-Private Partnership (PPP) with Inflammatix (unrestricted research grant to UMCG); a grant from Health Holland Public-Private Partnership (PPP) with Octapharma (unrestricted research grant to UMCG); He is unpaid board member of Dutch SepsisNet society (non-profit organization).

**Vladimir Černý** discloses honoraria for lectures from Octapharma and Astra Zeneca; He is unpaid president of the Czech Society of Anesthesiology and Intensive Care.

**Renata Curić Radivojević** has nothing to disclose.

**Ken Dewitte** has nothing to disclose.

**Matthias Gründling** discloses a grant from the German Federal Ministry of Health to the SepsisDialog/University of Greifswald for #DeutschlandErkenntSepsis; he also discloses payment and honoraria for lectures from Gradientech, bioMérieux, Pfizer, Shionogi, Sysmex, Becton Dickinson and Roche Diagnostics to SepsisDialog/University of Greifswald. He is board member of Deutschland Erkennt Sepsis and member of the Advisory Board of InfectoGnostics (Jena).

**Johanna Hästbacka** discloses a steering board membership of CRIC (Collaboartion for Research in Intensive Care, unpaid Chaiperson of the Finnish Sepsis Society and minor stock options of Orion Pharma).

**Said Laribi** has nothing to disclose.

**Annmarie Lassen** has nothing to disclose.

**Konstantin Lebedinskii** has nothing to disclose.

**Jan Máca** has nothing to disclose.

**Manu L.N.G. Malbrain** discloses grants from Baxter, Potrero Medical, Sentinel Medical, Medcaptain, Lexin Maltron and Grifols; consulting fees and honoraria from Baxter, Medcaptain, BD, Getinge, Fresenius-Kabi, Sentinel Medical, Medamen, LynxCare and GrifolsCytosorbents, Nestlé, PeerVoice, GE Healthcare and Medtronic; he discloses support for attending meetings from Sendinel Medical, Cytosorbents and Getinge; he discloses patents planned from Getinge Cimon and participation on data safety monitoring boards from Getinge, Baxter and Sentinel; he is president of the International Fluid Academy, treasurer of the Abdominal Compartment Society, specialist of the ESAIC and member of Global Sepsis Innovations Platform Group of the Globals Sepsis Alliance and member of the Sepsis Expert Group in Belgium; he also discloses stock options of Sentinal Medical Technoloigies, Potrero Medical and Serenno Medical.

**Gianpaola Monti** has nothing to disclose.

**Marlies Ostermann** is member of the Executive Committee of the European Society of Intensive Care Medicine and member of the Intensive Care Society UK and member of the Surviving Sepsis Campaign guideline panel.

**Michael Osthoff** discloses grants from Swiss National Science Foundation (project grant COVID-19 and TAVR); Pharming Technologies B.V. (unrestricted research grant) and Avelo (project grant breath collector); Consulting fees from Pharming Biotechnologies B.V. paid to his institution, lecture honoraria from GSK, payment for expert testimony from Pierre Fabre and support to attend ECCMID 2023 from Tillots Pharma, he also discloses receipt of equipment from Pharming Biotechnologies B.V.

**José Artur Paiva** has nothing to disclose.

**Michela Sabbatucci** has nothing to disclose.

**Jakub Śmiechowicz** has nothing to disclose.

**Mihai Gabriel Ştefan** discloses honoraria for lectures from Vifor Pharma and Takeda Pharmaceuticals.

**Marcus Vollmer** has nothing to disclose.

**Natalija Vukovic** has nothing to disclose.

**Kyriakos Zaragkoulias** discloses his membership in the Norwegian Society of Clinical Microbiology and his unpaid role as board member (representative for ESCMID Global).

**Konrad Reinhart** discloses holding shares from InflaRx NV, which is based in Jena, Germany and listed at NASDQ; he is the Founding President of the Global Sepsis Alliance and member of the steering committee of the European Sepsis Alliance.

**Adam Linder** has nothing to disclose.

**Daniela Filipescu** is Deputy chair of the European Sepsis Alliance (ESA) and representative of the European Society of Anaesthesiology and Intensive Care (ESAIC) to the ESA, both unpaid, she ist president of the World Federation of Societies of Anaesthesiologists (WFSA) and discloses a grant from CSL Vifor paid to the WFSA and honoraria from CSL Behring paid to the WFSA and travel support from CSL Behring, she ist scientific coordinator of the national congress of the Romanian Society of Anaesthesiology and Intensive Care.

**Ricard Ferrer** discloses consulting fees from Inotrem, Pfizer and Cytosorbent, he also discloses honoraria for lectures from Shionogi, MSD, Gilead, Menarini, Thermofisher, Viatris, AOP and Grifols.

**Evgeny A. Idelevich** discloses institutional grants from the German Federal Ministry of Education and Research (BMBF), from the Federal state Mecklenburg-Western Pomerania through the European Regional Development Fund (ERDF), as well as from MetaSystems Hard & Software GmbH, Bruker and Shionogi; inventor remuneration for patent licenses–3 patent applications licensed from the University of Münster to Bruker; consulting fees for expert role in the quality assurance procedure for the diagnosis, treatment, and follow-up care of sepsis from Institute for Quality Assurance and Transparency in Health Care (IQTIG); support for congress travel from Bruker; honoraria for educational lectures from Shionogi, Pfizer, BD.

## Appendix A Supplementary data

Supplemental Figures and Tables

ESC Survey-total
