# Supplementary material for: Blood culture practices and microbiological capacity for sepsis diagnostics in Europe (2021–2022): a cross-sectional analysis of the European Sepsis Care Survey
Source: Lancet Reg Health Eur. 2025 Dec 18;62:101570. doi: 10.1016/j.lanepe.2025.101570 (PMC12771334; doi:10.1016/j.lanepe.2025.101570)
Supplement: Supplemental Figures and Tables [file mmc1.docx]

Supplement to

**Blood Culture Practices and Microbiological Capacity for Sepsis Diagnostics in Europe (2021-2022): a cross-sectional analysis of the European Sepsis Care Survey**

**Corresponding Author:**

Dr. Christian S. Scheer, MD

University Medicine Greifswald

Department of Anaesthesiology, Intensive Care Medicine,

Emergency Medicine and Pain Medicine

Ferdinand-Sauerbruch-Straße 17475 Greifswald, Germany

christian.scheer@med.uni-greifswald.de

+49 3834 86 58 01

**Development, Harmonization and Pretesting of the ESCS**The ESCS, a cross-sectional questionnaire, was designed and harmonized by a multiprofessional steering committee led by the European Sepsis Alliance and peer- reviewed by the scientific committees of four European professional scientific societies (see below). The survey was available in English, German, Polish, Romanian, Russian, and Turkish. Technical and content-related pretesting was conducted by steering committee members and national coordinators.

**Handling of confidential information and data protection**

All data of the ESCS were handled confidentially. This was guaranteed before the study began. The questionnaire was conducted by using the survey software LamaPoll. LamaPoll meets the requirements of the European General Data Protection Regulation (EU GDPR) and was DIN ISO 27001 certified. The host was located in Germany. Data were stored at University Medicine Greifswald. Data analysis was conducted at University Medicine Greifswald without knowledge of the individual hospital locations and participants.

**Steering committee members:**

Adam Linder, Sweden (Chair ESA working group

research)

Christian S. Scheer, Germany (Principal investigator)

Daniela Filipescu (Vice-Chair of ESA)

Evangelos Giamarellos-Bourboulis, Greece (Chair of ESA)

Evgeny A. Idelevich, Germany (Representative ESCMID / ESGBIES)

Gabriella Bottari (Representative ESPNIC)

Konrad Reinhart, Germany (Founding president GSA)

Manu LNG Malbrain (Representative IFA)

Marlies Ostermann (Representative ICS UK)

Matthias Gründling, Germany (Quality management project Sepsisdialog Germany)

Ricard Ferrer (Representative ESICM)

Said Laribi (Representative EUSEM)

**Peer-reviewing scientific societies:**

The questionnaire of the ESCS was peer-reviewed by the scientific committees of ESAIC, ESCMID / ESGBIES, ESICM and ICS UK.

**Endorsing societies:**

The ESCS was endorsed by the ESAIC, ESCMID / ESGBIES, ESICM, ICS UK, ESPNIC, ESS, EUSEM and IFA.

ESA - European Sepsis Alliance

ESAIC - European Society of Anaesthesiology and Intensive Care

ESCMID / ESGBIES - Study Group for Bloodstream Infections, Endocarditis and Sepsis of the European Society of Clinical Microbiology and Infectious Diseases

ESICM - European Society of Intensive Care Medicine

ESPNIC - European Society of Paediatric and Neonatal Intensive Care

ESS - European Shock Society

EUSEM - European Society for Emergency Medicine

GSA - Global Sepsis Alliance

ICS - UK Intensive Care Society United Kingdom

IFA - International Fluid Academy

**sTable 1. Details about the participants in the hospitals (n=907)**

| **Position of the participants in the hospitals** | | |
| --- | --- | --- |
| Hospital director | 54 (6%) | |
| Head or deputy head of department | 403 (44%) | |
| Consultant | 350 (39%) | |
| Other (doctors, residents, nurses, etc.) | 100 (11%) | |
| **Profession/specialty of the participants in the hospitals** | | |
| Emergency medicine | 59 (6%) | Respondents consulted colleagues from the emergency department in 17%, from the wards in 11%, from the intensive care units in 17% and from the microbiology in 15% for detailed information. |
| Medicine, internal medicine, non-operative disciplines) | 55 (6%) |  |
| Anaesthesia, intensive care medicine, reanimatology | 339 (37%) |  |
| Infectious diseases | 64 (7%) |  |
| Surgery | 32 (4%) |  |
| Intensive care medicine | 200 (22%) |  |
| Hospital director | 54 (6%) |  |
| Microbiology | 15 (2%) |  |
| Other (i.e. infection prevention, antibiotic stewardship teams, quality management officers) | 89 (10%) |  |

**sTable 2. Participating countries, included hospitals and represented bed capacity per country**

The European Sepsis Care Survey was disseminated by email invitations as well as announcements and calls from scientific societies and professional networks. Therefore, it was not possible to calculate a classical response rate, which requires the number of participants as the numerator and the number of the individuals contacted as the denumerator. Moreover, comparable information about the number of hospitals in each country was not available. Therefore, it was not possible to calculate a response rate per country. However, we had access to data on hospital bed capacity in each country (based on OECD and EUROSTAT sources), as well as the number of beds covered in our study (the beds in each of the participating hospitals). Based on this information, we calculated a “participation rate” or “bed coverage” per country using the bed capacity in the hospitals in the study as the numerator and the total bed capacity in the country as the denumerator.

Bed capacity according to the Organization for Economic Co-operation and Development (OECD) and the European Statistics Office (EUROSTAT), the European Union has 1,731,000 hospital beds dedicated to acute care.

| **Country** | **Included hospitals** | **Bed capacity in the included hospitals** | **Total bed capacity per country^1^** | **Bed capacity in the hospitals in the study**  **Total bed capacity in the country** |
| --- | --- | --- | --- | --- |
| Sweden | 35 | 14179 | 15113 | 93·8% |
| Iceland | 1 | 657 | 849 | 77·4% |
| Croatia | 21 | 10965 | 14286 | 76·8% |
| Malta | 1 | 1243 | 1640 | 75·8% |
| Finland | 17 | 9698 | 13090 | 74·1% |
| Czechia | 41 | 25549 | 43630 | 58·6% |
| Slovenia | 3 | 3577 | 6965 | 51·4% |
| Netherlands | 27 | 18556 | 38779 | 47·9% |
| Denmark | 4 | 3622 | 11070 | 32·7% |
| Norway | 6 | 3467 | 10852 | 31·9% |
| Ireland | 14 | 4197 | 13626 | 30·8% |
| Luxembourg | 1 | 579 | 2042 | 28·4% |
| Belgium | 23 | 14999 | 56871 | 26·4% |
| Greece | 17 | 9792 | 38611 | 25·4% |
| Italy | 63 | 38170 | 154151 | 24·8% |
| Spain | 51 | 29123 | 119490 | 24·4% |
| Germany | 245 | 116489 | 483606 | 24·1% |
| Portugal | 17 | 8206 | 34456 | 23·8% |
| Estonia | 1 | 800 | 3767 | 21·2% |
| Türkiye | 71 | 51633 | 248050 | 20·8% |
| Romania | 36 | 19315 | 106067 | 18·2% |
| Switzerland | 8 | 5096 | 30591 | 16·7% |
| Serbia | 9 | 4565 | 28986 | 15·7% |
| Poland | 55 | 25056 | 166338 | 15·1% |
| United Kingdom | 22 | 13950 | 94827 | 14·7% |
| France | 27 | 23976 | 195317 | 12·3% |
| Austria | 4 | 4992 | 45067 | 11·1% |
| Rep. of Moldova | 3 | 1480 | 20221 | 7·3% |
| Bulgaria | 5 | 3218 | 45803 | 7·0% |
| Cyprus | 1 | 140 | 2813 | 5·0% |
| Russian Fed. | 68 | 44235 | 1172800 | 3·8% |
| Hungary | 2 | 1350 | 41617 | 3·2% |
| Albania | 1 | 193 | 8253 | 2·3% |
| Slovakia | 1 | 126 | 26270 | 0·5% |
| Belarus | 1 | 183 | 101610 | 0·2% |
| Ukraine | 3 | 1860 | 312996 | 0·6% |
| North Macedonia | 2 | 51 | 6258 | 0·8% |

1 According to OECD data (https://stats.oecd.org/index.aspx?queryid=30182#) and EUROSTAT 2019 (https://ec.europa.eu/eurostat/statistics-

explained/index.php?title=Healthcare_resource_statistics_-_beds) (reference year 2019/2020 or most recent); data about the Russian Federation were reported by the national coordinator for the Russian Federation.

**sTable 3. Characteristics of included hospitals**

| **Hospital size** | **Number** | **%** |
| --- | --- | --- |
| Total | 907 | 100 |
| Hospitals 0-250 beds | 260 | 28·7 |
| Hospitals 251-500 beds | 268 | 29·5 |
| Hospitals 501-750 beds | 140 | 15·4 |
| Hospitals 751-1000 beds | 118 | 13·0 |
| Hospitals >1000 beds | 121 | 13·3 |
|  |  |  |
| University hospital or teaching hospital | 312 | 34·4 |
| General or community hospital | 549 | 60·5 |
| Independent or private hospital | 46 | 5·1 |

**sFigure 1. Represented bed capacity in the study**

The sample represents 388,574 out of 1,681,334 (23·1%) of all acute care beds in the 26 European Union countries included in the study.


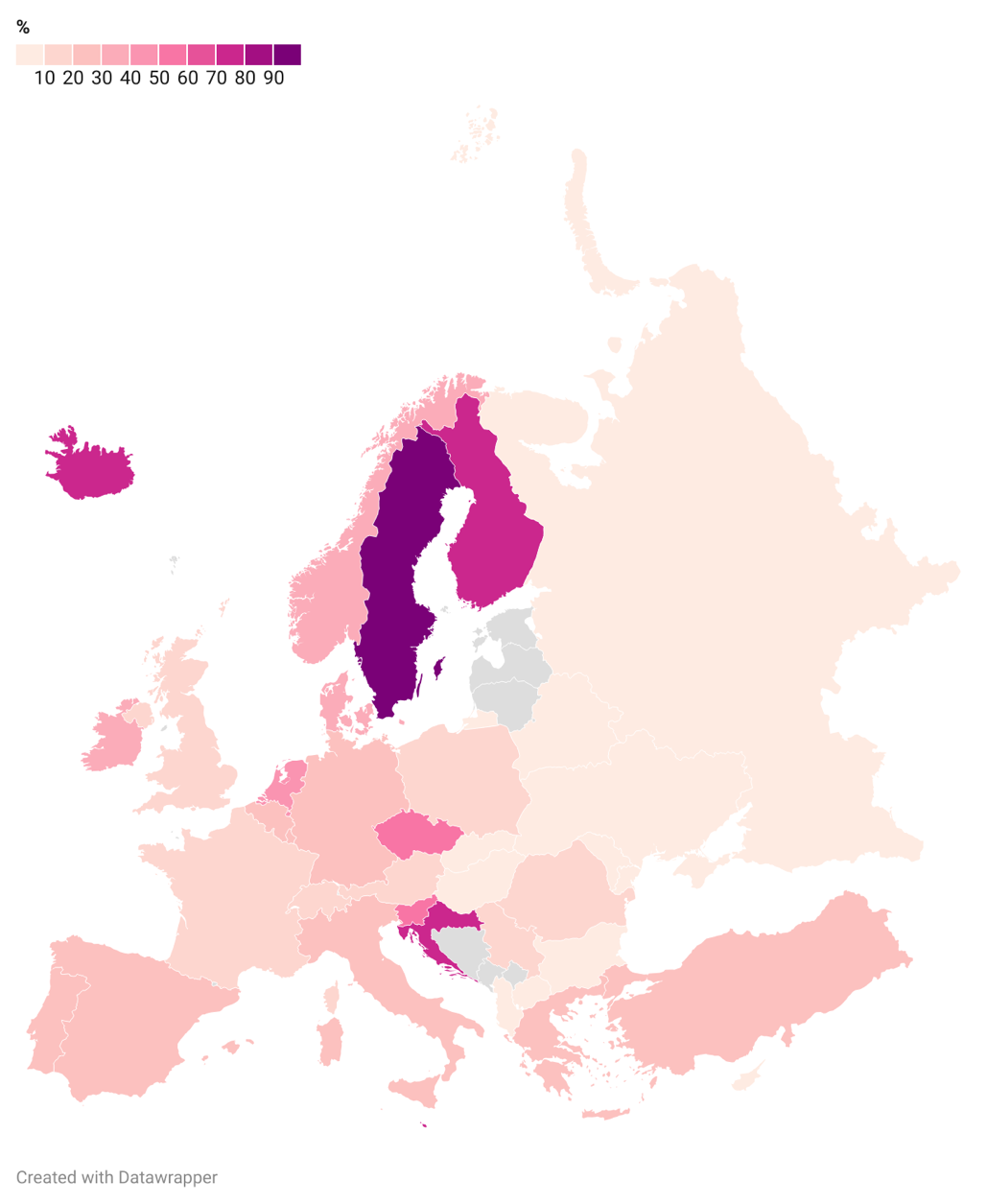

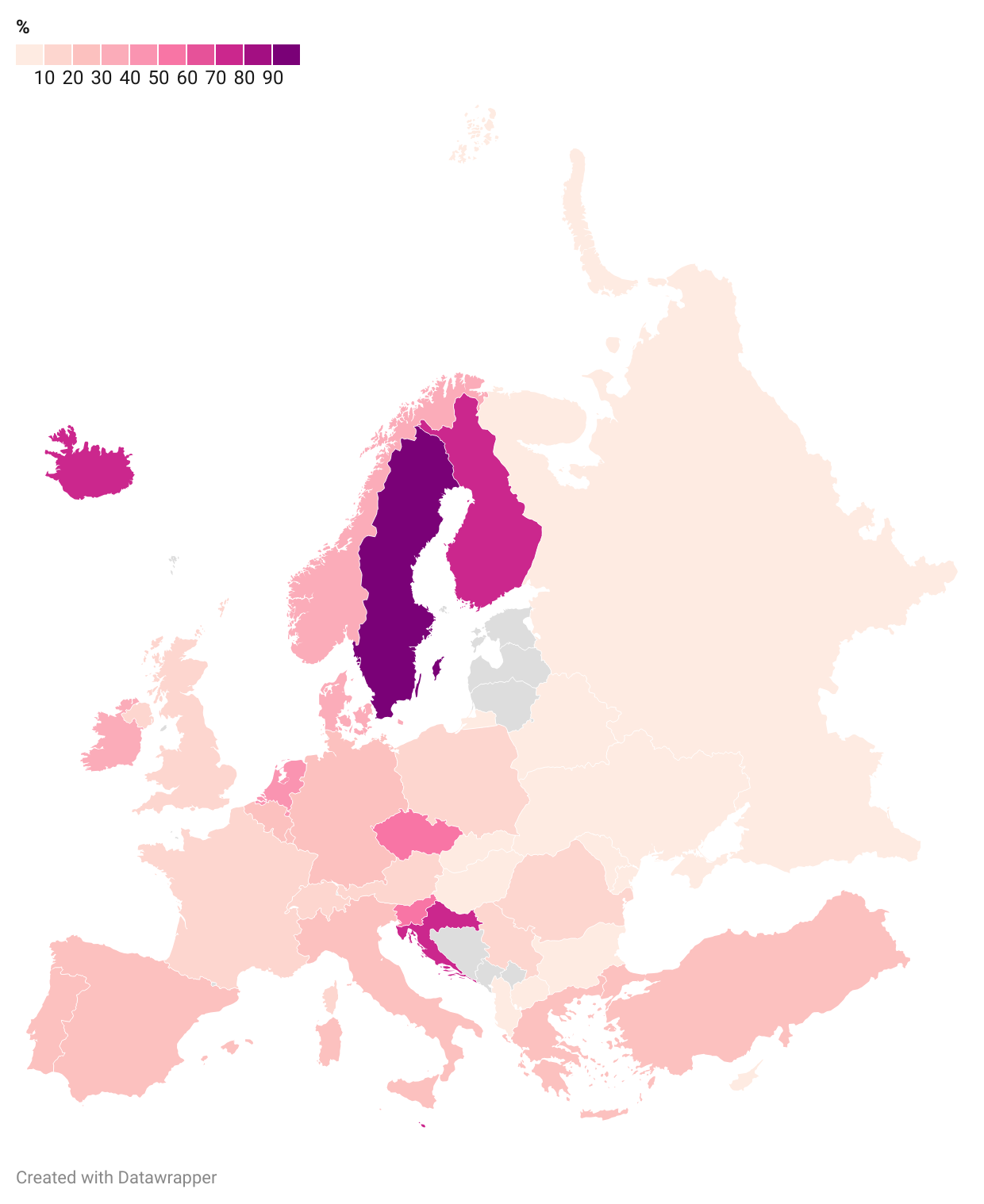


**sTable 4. Preferred and alternative sites for blood culture collection**

|  | Direct venipuncture at two different sites | | | Direct venipuncture at one site | | From new (≤24h) catheters (arterial or central) | | From old (>24h) catheter (arterial or central) | | From old (>24h) catheter on suspicion of catheter infection | |
| --- | --- | --- | --- | --- | --- | --- | --- | --- | --- | --- | --- |
|  | n | % | n | | % | n | % | n | % | n | % |
| **Emergency departments (n=735)** |  | | | | | | | | | | |
| preferred (primary) collection | 461 | 62·7 | 260 | | 35·4 | 151 | 20·5 | 90 | 12·2 | 323 | 43·9 |
| first alternative collection | 89 | 12·1 | 290 | | 39·5 | 203 | 27·6 | 62 | 8·4 | 71 | 9·7 |
| second alternative collection | 45 | 6·1 | 79 | | 10·7 | 197 | 26·8 | 97 | 13·2 | 51 | 6·9 |
| third alternative collection | 13 | 1·8 | 26 | | 3·5 | 30 | 4·1 | 141 | 19·2 | 72 | 9·8 |
| never used | 97 | 13·2 | 50 | | 6·8 | 124 | 16·9 | 316 | 42·9 | 188 | 25·6 |
| not selected | 30 | 4·1 | 30 | | 4·1 | 30 | 4·1 | 30 | 4·1 | 30 | 4·1 |
| **Wards (n=802)** | | | | | | | | | | | |
| preferred (primary) collection | 513 | 64·0 | 269 | | 33·5 | 143 | 17·8 | 108 | 13·5 | 321 | 40·0 |
| first alternative collection | 119 | 14·8 | 349 | | 43·5 | 196 | 24·4 | 90 | 11·2 | 98 | 12·2 |
| second alternative collection | 36 | 4·5 | 75 | | 9·4 | 230 | 28·7 | 110 | 13·7 | 58 | 7·2 |
| third alternative collection | 19 | 2·4 | 27 | | 3·4 | 47 | 5·9 | 171 | 21·3 | 77 | 9·6 |
| never used | 88 | 11·0 | 55 | | 6·9 | 159 | 19·8 | 296 | 36·9 | 221 | 27·6 |
| not selected | 27 | 3·4 | 27 | | 3·4 | 27 | 3·4 | 27 | 3·4 | 27 | 3·4 |
| **Intensive care units (n=756)** | | | | | | | | | | | |
| preferred (primary) collection | 518 | 68·5 | 198 | | 26·2 | 260 | 34·4 | 138 | 18·3 | 374 | 49·5 |
| first alternative collection | 105 | 13·9 | 338 | | 44·7 | 219 | 29·0 | 96 | 12·7 | 83 | 11·0 |
| second alternative collection | 33 | 4·4 | 87 | | 11·5 | 170 | 22·5 | 104 | 13·8 | 54 | 7·1 |
| third alternative collection | 24 | 3·2 | 40 | | 5·3 | 43 | 5·7 | 155 | 20·5 | 72 | 9·5 |
| never used | 68 | 9·0 | 85 | | 11·2 | 56 | 7·4 | 255 | 33·7 | 165 | 21·8 |
| not selected | 8 | 1·1 | 8 | | 1·1 | 8 | 1·1 | 8 | 1·1 | 8 | 1·1 |

**sTable 5. Number of blood culture sets in emergency departments, wards, and intensive care units**

|  | **One blood culture set** | | **Two blood culture set** | | **Three blood culture set** | | **Four blood culture set** | | **More than four blood culture sets** | |
| --- | --- | --- | --- | --- | --- | --- | --- | --- | --- | --- |
|  | n | % | n | % | n | % | n | % | n | % |
| **Emergency departments (n=704)** | | | | | | | | | | |
| always, 100% of the blood culture samplings | 270 | 38·4 | 218 | 31·0 | 13 | 1·8 | 8 | 1·1 | 7 | 1·0 |
| in more than 75% of the blood culture samplings | 54 | 7·7 | 193 | 27·4 | 37 | 5·3 | 7 | 1·0 | 7 | 1·0 |
| 50-75% of the blood culture samplings | 43 | 6·1 | 77 | 10·9 | 31 | 4·4 | 14 | 2·0 | 8 | 1·1 |
| in 25-50% of the blood culture samplings | 47 | 6·7 | 51 | 7·2 | 58 | 8·2 | 15 | 2·1 | 5 | 0·7 |
| in less than 25% of the blood culture samplings | 149 | 21·2 | 69 | 9·8 | 235 | 33·4 | 108 | 15·3 | 73 | 10·4 |
| never (0%) of the blood culture samplings | 141 | 20·0 | 96 | 13·6 | 330 | 46·9 | 552 | 78·4 | 604 | 85·8 |

| **Wards (n=775)** | | | | | | | | | | |
| --- | --- | --- | --- | --- | --- | --- | --- | --- | --- | --- |
| always, 100% of the blood culture samplings | 314 | 40·5 | 218 | 28·1 | 14 | 1·8 | 9 | 1·2 | 9 | 1·2 |
| in more than 75% of the blood culture samplings | 82 | 10·6 | 249 | 32·1 | 38 | 4·9 | 9 | 1·2 | 6 | 0·8 |
| 50-75% of the blood culture samplings | 60 | 7·7 | 88 | 11·4 | 53 | 6·8 | 13 | 1·7 | 7 | 0·9 |
| in 25-50% of the blood culture samplings | 47 | 6·1 | 49 | 6·3 | 62 | 8·0 | 19 | 2·5 | 5 | 0·6 |
| in less than 25% of the blood culture samplings | 149 | 19·2 | 83 | 10·7 | 236 | 30·5 | 141 | 18·2 | 88 | 11·4 |
| never (0%) of the blood culture samplings | 123 | 15·9 | 88 | 11·4 | 372 | 48·0 | 584 | 75·4 | 660 | 85·2 |

| **Intensive care units (n=748)** | | | | | | | | | | |
| --- | --- | --- | --- | --- | --- | --- | --- | --- | --- | --- |
| always, 100% of the blood culture samplings | 261 | 34·9 | 293 | 39·2 | 39 | 5·2 | 11 | 1·5 | 11 | 1·5 |
| in more than 75% of the blood culture samplings | 64 | 8·6 | 225 | 30·1 | 78 | 10·4 | 12 | 1·6 | 7 | 0·9 |
| 50-75% of the blood culture samplings | 35 | 4·7 | 56 | 7·5 | 83 | 11·1 | 20 | 2·7 | 9 | 1·2 |
| in 25-50% of the blood culture samplings | 42 | 5·6 | 49 | 6·6 | 74 | 9·9 | 42 | 5·6 | 16 | 2·1 |
| in less than 25% of the blood culture samplings | 135 | 18·0 | 59 | 7·9 | 221 | 29·5 | 186 | 24·9 | 127 | 17·0 |
| never (0%) of the blood culture samplings | 211 | 28·2 | 66 | 8·8 | 253 | 33·8 | 477 | 63·8 | 578 | 77·3 |

**sTable 6. Transfer priority of blood cultures after collection**

|  | **Emergency departments**  **On-site microbiology**  **(n=327)** | | **Emergency departments External microbiology**  **(n=383)** | |
| --- | --- | --- | --- | --- |
|  | n | % | n | % |
| They are sent immediately after being taken | 250 | 76·5 | 245 | 64·0 |
| They are sent at set times throughout the day (e.g. morning shift, afternoon shift, night shift) irrespective of extraction time | 40 | 12·2 | 96 | 25·1 |
| They are stored in the extraction department and collected by personnel from the laboratory as they become available | 23 | 7·0 | 23 | 6·0 |
| I don't know | 14 | 4·3 | 19 | 5·0 |

|  | **Wards**  **On-site microbiology**  **(n=371)** | | **Wards External microbiology**  **(n=404)** | |
| --- | --- | --- | --- | --- |
|  | n | % | n | % |
| They are sent immediately after being taken | 277 | 74·7 | 248 | 61·4 |
| They are sent at set times throughout the day (e.g. morning shift, afternoon shift, night shift) irrespective of extraction time | 58 | 15·6 | 113 | 28·0 |
| They are stored in the extraction department and collected by personnel from the laboratory as they become available | 29 | 7·8 | 30 | 7·4 |
| I don't know | 7 | 1·9 | 13 | 3·2 |

|  | **Intensive care units**  **On-site microbiology**  **(n=358)** | | **Intensive care units**  **External microbiology**  **(n=390)** | |
| --- | --- | --- | --- | --- |
|  | n | % | n | % |
| They are sent immediately after being taken | 296 | 82·7 | 279 | 71·5 |
| They are sent at set times throughout the day (e.g. morning shift, afternoon shift, night shift) irrespective of extraction time | 36 | 10·1 | 89 | 22·8 |
| They are stored in the extraction department and collected by personnel from the laboratory as they become available | 23 | 6·4 | 21 | 5·4 |
| I don't know | 3 | 0·8 | 1 | 0·3 |

**sTable 7. External microbiology laboratories depending on hospital size**

|  | **External microbiology** | **On-site microbiology** | **Total** | **External microbiology**  **%** |
| --- | --- | --- | --- | --- |
| 0-250 beds | 148 | 72 | 220 | 67·27 |
| 251-500 beds | 138 | 115 | 253 | 54·55 |
| 501-750 beds | 64 | 69 | 133 | 48·12 |
| 751-1000 beds | 45 | 69 | 114 | 39·47 |
| >1000 beds | 41 | 77 | 118 | 34·75 |

**sFigure 2. Turn-around times of blood cultures**

Bars represent the cumulative proportion of blood culture results at various time intervals from collection on wards and intensive care units.

**Preliminary result, e.g. Gram stain microscopy**

**Final result including pathogen identification and antimicrobial susceptibility testing**
