# Supplementary material for: Blood culture practices and microbiological capacity for sepsis diagnostics in Europe (2021–2022): a cross-sectional analysis of the European Sepsis Care Survey
Source: Lancet Reg Health Eur. 2025 Dec 18;62:101570. doi: 10.1016/j.lanepe.2025.101570 (PMC12771334; doi:10.1016/j.lanepe.2025.101570)
Supplement: ESC Survey-total [file mmc2.pdf]

European Sepsis Alliance

European Sepsis Care Survey

Thank you for participating!

*Officially endorsed by the **European Society of Anaesthesiology and Intensive Care (ESAIC)**, the **European Society of Intensive Care Medicine (ESICM)**, the **European Society of Clinical Microbiology and Infectious Diseases (ESCMID)** and the **European Shock Society (ESS)***

The EUROPEAN SEPSIS CARE SURVEY

Responsible persons who are filling this survey will become members of the European Sepsis Care study group and will be acknowledged as collaborators and PubMed listed.
In case of questions, please send an email to sepsissurvey@med.uni-greifswald.de

The survey explores potential confidential hospital information. If necessary, local hospital coordinators
should obtain approval to participate in the survey prior
to participation.This may differ in different
countries. It is the responsibility of the respondents to check whether local approval is necessary.

The questionnaire does NOT ask for your name and does NOT ask patient data. Conclusions about individual hospitals will NOT be drawn and NOT published.

All data will be handled confidentially.

General Questions

Please liaise with colleagues if you cannot answer the questions.

You can share the questionaire by sharing the link in your browser.

Type of your hospital

- General/community hospital
- University hospital
- Independent hospital
- Other (please specify)

How many beds does your hospital have?

Please tick the box that describes your position

- ◯ Hospital or medical director
- ◯ Head of .... (please specify your subject area)
- ◯ Consultant (please specify your subject area)
- ◯ Other (please specify)

General Questions

☆ How is sepsis defined in your hospital?

Multiple answers possible.

- [ ] Systemic inflammatory response syndrome (SIRS) criteria due to infection.
- [ ] New organ failure as evidenced by an increase in SOFA score by ≧2 (Sequential organ failure assessment) due to infection.
- [ ] Other (please describe)
- [ ] I don't know

European Sepsis Alliance

European Sepsis Care Survey

Thank you for participating!

*Officially endorsed by the **European Society of Anaesthesiology and Intensive Care (ESAIC)**, the **European Society of Intensive Care Medicine (ESICM)**, the **European Society of Clinical Microbiology and Infectious Diseases (ESCMID)** and the **European Shock Society (ESS)***

General Questions

What specialties does your hospital have?

Multiple answer possible.

- [ ] **Emergency department**
- [ ] **Intensive care unit**
- [ ] **General surgery**
- [ ] **Internal medicine**
- [ ] Cardiology
- [ ] Neurology
- [ ] Traumatology
- [ ] Paediatrics
- [ ] Gynaecology
- [ ] Urology
- [ ] Cardiac surgery
- [ ] Neurosurgery
- [ ] Infectious diseases
- [ ] **Clinical chemistry laboratory**
- [ ] **Microbiological laboratory**
- [ ] **External microbiological laboratory**
- [ ] **Radiology**
- [ ] Infection control department
- [ ] Others (please specify)

European Sepsis Alliance

European Sepsis Care Survey

Thank you for participating!

*Officially endorsed by the **European Society of Anaesthesiology and Intensive Care (ESAIC)**, the **European Society of Intensive Care Medicine (ESICM)**, the **European Society of Clinical Microbiology and Infectious Diseases (ESCMID)** and the **European Shock Society (ESS)***

General Questions

Do you have a medical emergency team or critical care outreach team in your hospital?

A medical emergency team refers to a team for the evaluation of critically ill patientsor patients at risk. This includes a nurse-lead critical care outreach team for example. Note: it is not a cardiopulmonary resuscitation team

- ○ Yes
- ○ No
- ○ I don't know

European Sepsis Alliance

European Sepsis Care Survey

Thank you for participating!

*Officially endorsed by the **European Society of Anaesthesiology and Intensive Care (ESAIC)**, the **European Society of Intensive Care Medicine (ESICM)**, the **European Society of Clinical Microbiology and Infectious Diseases (ESCMID)** and the **European Shock Society (ESS)***

General Questions

What kind of service does your clinical chemistry laboratory provide?

- ◯ 24/7 service
- ◯ Service only in main working hours
- ◯ We use an external clinical chemistry laboratory

European Sepsis Alliance

European Sepsis Care Survey

Thank you for participating!

*Officially endorsed by the **European Society of Anaesthesiology and Intensive Care (ESAIC)**, the **European Society of Intensive Care Medicine (ESICM)**, the **European Society of Clinical Microbiology and Infectious Diseases (ESCMID)** and the **European Shock Society (ESS)***

General Questions

What kind of service does your microbiological laboratory provide?

	Monday - Friday working hours	Monday - Friday after working hours emergency service on demand	Monday - Friday 24h service	Weekend and holidays limited service	Weekend and holidays emergency service on demand	Weekend holidays 24h service
Blood culture incubation	☐	☐	☐	☐	☐	☐
Blood culture gram staining	☐	☐	☐	☐	☐	☐
Pathogen identification from blood cultures	☐	☐	☐	☐	☐	☐
Antibiotic susceptibility testing from blood cultures	☐	☐	☐	☐	☐	☐
Notification about blood culture results	☐	☐	☐	☐	☐	☐

European Sepsis Alliance

European Sepsis Care Survey

Thank you for participating!

*Officially endorsed by the **European Society of Anaesthesiology and Intensive Care (ESAIC)**, the **European Society of Intensive Care Medicine (ESICM)**, the **European Society of Clinical Microbiology and Infectious Diseases (ESCMID)** and the **European Shock Society (ESS)***

General Questions

Do you have a rapid microbiological testing service available at your hospital?

Multiple answers possible.

- [ ] Yes, we have rapid identification from positive blood cultures (e.g., by PCR, direct MALDI-TOF, MALDI-TOF after short incubation)
- [ ] Yes, we have rapid antimicrobial susceptibility testing directly from positive blood cultures
- [ ] No
- [ ] I don't know

European Sepsis Alliance

European Sepsis Care Survey

Thank you for participating!

*Officially endorsed by the **European Society of Anaesthesiology and Intensive Care (ESAIC)**, the **European Society of Intensive Care Medicine (ESICM)**, the **European Society of Clinical Microbiology and Infectious Diseases (ESCMID)** and the **European Shock Society (ESS)***

General Questions

Are you satisfied with the diagnostic service provided by your microbiological laboratory for sepsis patients?

Please select...

☆ ☆ ☆ ☆ ☆

European Sepsis Alliance

European Sepsis Care Survey

Thank you for participating!

*Officially endorsed by the **European Society of Anaesthesiology and Intensive Care (ESAIC)**, the **European Society of Intensive Care Medicine (ESICM)**, the **European Society of Clinical Microbiology and Infectious Diseases (ESCMID)** and the **European Shock Society (ESS)***

General Questions

How could your microbiological diagnostic service improve?

Multiple answers possible

- [ ] Microbiological diagnostics of sepsis should be more rapid
- [ ] Microbiological diagnostics of sepsis should be more precise
- [ ] More specific advice (e.g. antibiotic choice, dosage, duration)
- [ ] 24/7 microbiological service
- [ ] None, I am satisfied

European Sepsis Alliance

European Sepsis Care Survey

Thank you for participating!

Officially endorsed by the ***European Society of Anaesthesiology and Intensive Care (ESAIC)****, the* ***European Society of Intensive Care Medicine (ESICM)****, the* ***European Society of Clinical Microbiology and Infectious Diseases (ESCMID)*** *and the* ***European Shock Society (ESS)***

General Questions

What kind of service does your radiology department provide?

CT= computer tomography; MR = magnetic resonance imaging

- [ ] CT / MR scans 24/7
- [ ] CT / MR scans are only available in main working hours
- [ ] Patients have to be transferred for CT / MR scans to another hospital
- [ ] No CT / MR scanner available (e.g. only chest x ray)
- [ ] Interventional radiology

European Sepsis Alliance

European Sepsis Care Survey

Thank you for participating!

*Officially endorsed by the **European Society of Anaesthesiology and Intensive Care (ESAIC)**, the **European Society of Intensive Care Medicine (ESICM)**, the **European Society of Clinical Microbiology and Infectious Diseases (ESCMID)** and the **European Shock Society (ESS)***

General Questions

Do you have a standard operating procedure or hospital guideline for antimicrobial therapy for septic patients?

- ◯ Yes
- ◯ No
- ◯ I don't know

European Sepsis Alliance

European Sepsis Care Survey

Thank you for participating!

Officially endorsed by the ***European Society of Anaesthesiology and Intensive Care (ESAIC)****, the* ***European Society of Intensive Care Medicine (ESICM)****, the* ***European Society of Clinical Microbiology and Infectious Diseases (ESCMID)*** *and the* ***European Shock Society (ESS)***

General Questions

Do you have a guideline or protocol for taking blood cultures?

- ◯ Yes
- ◯ No
- ◯ I don't know

European Sepsis Alliance

European Sepsis Care Survey

Thank you for participating!

*Officially endorsed by the **European Society of Anaesthesiology and Intensive Care (ESAIC)**, the **European Society of Intensive Care Medicine (ESICM)**, the **European Society of Clinical Microbiology and Infectious Diseases (ESCMID)** and the **European Shock Society (ESS)***

General Questions

How do you track the blood culture sample, from collection to start of incubation, until the final report?

- ◯ We don't have such a system in place
- ◯ The status of each blood culture can be seen with delay in our laboratory/hospital information system
- ◯ The status of each blood culture can be seen in real-time in our laboratory/hospital information system
- ◯ Other (please specify)
- ◯ I don't know

European Sepsis Alliance

European Sepsis Care Survey

Thank you for participating!

Officially endorsed by the ***European Society of Anaesthesiology and Intensive Care (ESAIC)****, the* ***European Society of Intensive Care Medicine (ESICM)****, the* ***European Society of Clinical Microbiology and Infectious Diseases (ESCMID)*** *and the* ***European Shock Society (ESS)***

General Questions

Do you have an antibiotic stewardship team (ABS) at your hospital?

- ◯ Yes
- ◯ No
- ◯ I don't know

European Sepsis Alliance

European Sepsis Care Survey

Thank you for participating!

Officially endorsed by the ***European Society of Anaesthesiology and Intensive Care (ESAIC)****, the* ***European Society of Intensive Care Medicine (ESICM)****, the* ***European Society of Clinical Microbiology and Infectious Diseases (ESCMID)*** *and the* ***European Shock Society (ESS)***

General Questions

With regard to source control, which statements apply to your hospital?

Multiple answer possible

- [ ] Surgical source control is available 24/7 at our hospital.
- [ ] Surgical source control is only available in working hours (daytime).
- [ ] Source control by interventional radiology is available 24/7 at our hospital.
- [ ] Source control by interventional radiology is only available during working hours (daytime)
- [ ] Source control is not available at our hospital, patients must be transferred.
- [ ] I don't know

European Sepsis Alliance

European Sepsis Care Survey

Thank you for participating!

*Officially endorsed by the **European Society of Anaesthesiology and Intensive Care (ESAIC)**, the **European Society of Intensive Care Medicine (ESICM)**, the **European Society of Clinical Microbiology and Infectious Diseases (ESCMID)** and the **European Shock Society (ESS)***

General Questions

When needed, how do you prioritise source control compared to antibiotic treatment in septic patients?

- ◯ Source control is prioritized before antibiotics
- ◯ If patient receive antibiotics, source control is of secondary importance
- ◯ Source control is done in parallel with antibiotics
- ◯ I don't know

Questions about sepsis care in the EMERGENCY DEPARTMENT

How many patients do you treat usually per month in the emergency department?

[ ] patients / month

Please estimate the number of sepsis cases per month in the emergency department.

[ ] sepsis cases / month

Do you use a triage system or scores in the emergency department?

- ◯ Yes
- ◯ No
- ◯ I don't know

Please tick the triage systems and scores used in the emergency department

Multiple answers possible.

- [ ] Manchester triage system (MTS)
- [ ] Emergency Severity Index
- [ ] ABCDE
- [ ] Early warning scores (e.g. NEWS, MEWS)
- [ ] RETTS (Rapid Emergency Triage and Treatment System)
- [ ] STaRT (Simple Triage and rapid treatment)
- [ ] mSTaRT
- [ ] BASIC (Bleeding, Airway, Shock, Immobilisation after Classification)
- [ ] SOFA
- [ ] qSOFA
- [ ] Other (please describe) ______
- [ ] none

Do you have a protocol or standardized screening tool specifically for the recognition of sepsis in the emergency department?

- ( ) Yes
- ( ) No
- ( ) I don't know

Please select the criteria used to screen patients for sepsis in the

emergency department.

Multiple answers possible.
Please select all applicable criteria!

- ☐ Respiratory rate
- ☐ Temperature
- ☐ Mental alteration (e.g. Glasgow Coma Scale)
- ☐ Behavioural change
- ☐ Heart rate
- ☐ Blood pressure
- ☐ Urine output
- ☐ Lactate
- ☐ Organ dysfunction (e.g. mental status, cardiovascular system, respiration, renal function)
- ☐ qSOFA
- ☐ SOFA
- ☐ SIRS-criteria
- ☐ Early warning scores (e.g. NEWS, MEWS)
- ☐ White blood cells / Leucocytes
- ☐ Procalcitonin (PCT)
- ☐ C-reactive protein (CRP)
- ☐ IL-6
- ☐ Suspected infection
- ☐ Confirmed infection

- [ ] Other (Please describe)

Do you have a protocol, care pathway or bundle specifically for the management of sepsis in the emergency department?

- ○ Yes
- ○ No
- ○ I don't know

Please choose the actions included in your sepsis protocol, care pathway or bundle in the emergency department.

Multiple answers possible.

- [ ] Measure lactate level
- [ ] Obtain blood cultures before administration of antibiotics
- [ ] Administer broad-spectrum antibiotics
- [ ] Begin rapid administration of 30ml/kg of crystalloid for hypotension or lactate ≥4mmol/L
- [ ] Apply vasopressors if patient is hypotensive during or after fluid resuscitation to maintain MAP ≥65mmHg

Do you have a specified time frame for these actions?

- ◯ No
- ◯ 1h
- ◯ 2h
- ◯ 6h
- ◯ 12h
- ◯ 24h

Please select further actions, that are performed in the emergency department and the corresponding time frame.

Multiple answers possible.
Please select all applicable criteria!

					Time frame			
	Part of sepsis bundle	Part of sepsis protocol or pathway	Only on demand	Never	within 1-6 hours	within 12 hours	within 24 hours	no time frame
Physical examination	☐	☐	☐	☐	◯	◯	◯	◯
Blood testing (white blood cells, coagulation, renal and liver function)	☐	☐	☐	☐	◯	◯	◯	◯
SOFA score	☐	☐	☐	☐	◯	◯	◯	◯
CT scan for source identification	☐	☐	☐	☐	◯	◯	◯	◯
Ultrasound for source identification	☐	☐	☐	☐	◯	◯	◯	◯

Chest-X-ray for source identification	☐	☐	☐	☐	○	○	○	○
Microbiological sampling (e.g. urine, swabs, cerebrospinal fluid)	☐	☐	☐	☐	○	○	○	○
Catheterization / urine output measurement	☐	☐	☐	☐	○	○	○	○
Initiation of rapid source control (surgical or interventional)	☐	☐	☐	☐	○	○	○	○
Central venous line	☐	☐	☐	☐	○	○	○	○
ScvO2	☐	☐	☐	☐	○	○	○	○
Arterial line	☐	☐	☐	☐	○	○	○	○
Passive leg raising or fluid challenge	☐	☐	☐	☐	○	○	○	○
Cardiac output, stroke volume, pulse pressure measurement	☐	☐	☐	☐	○	○	○	○
Regular consultations with infectious diseases specialists	☐	☐	☐	☐	○	○	○	○
Procalcitonin guided anti-infective therapy	☐	☐	☐	☐	○	○	○	○
MRSA-PCR-testing	☐	☐	☐	☐	○	○	○	○
Other rapid microbiological testing (please specify)	☐	☐	☐	☐	○	○	○	○

Other actions (please specify)

Do you have point-of-care blood gas/lactate analytics in the emergency department?

- Yes
- No
- I don't know

When do you obtain blood cultures in the emergency department?

Multiple answer possible.

- They are obtained when patients have a fever
- They are obtained when there is shock
- There are obtained in any case of infection or suspicion of infection
- They are not routinely obtained
- Other (please specify)

Who takes the blood cultures in the emergency department?

Multiple answers possible

- ☐ Nursing staff
- ☐ Physicians
- ☐ Specialized phlebotomy staff
- ☐ Depends on the availability of staff

From what site do you collect blood cultures in the emergency department?

	preferred (primary) collection	first alternative collection	second alternative collection	third alternative collection	never used
Direct venepuncture at two different sites	○	○	○	○	○
Direct venepuncture at one site	○	○	○	○	○
From new (24h) catheters (arterial or central)	○	○	○	○	○
From old (>24h) catheter (arterial or central)	○	○	○	○	○
From old (>24h) catheter on suspicion of catheter infection	○	○	○	○	○

How many blood cultures do you take at the sepsis suspicion in the emergency department?

A set consists of 2 vials, one aerobic vial and one anaerobic vial

	always, 100% of the blood culture samplings	in more than 75% of the blood culture samplings	50-75% of the blood culture samplings	in 25-50% of the blood culture samplings	in less than 25% of the blood culture samplings	never (0%) of the blood culture samplings
1 set (2 vials)	◯	◯	◯	◯	◯	◯
2 sets (4 vials)	◯	◯	◯	◯	◯	◯
3 sets (6 vials)	◯	◯	◯	◯	◯	◯
4 sets (8 vials)	◯	◯	◯	◯	◯	◯
more than 4 sets	◯	◯	◯	◯	◯	◯

With what priority are blood cultures sent to the laboratory in the emergency department?

- ◯ They are sent immediately after being taken
- ◯ They are sent at set times during the day (e.g. morning shift, afternoon shift, night shift) irrespective of sampling time
- ◯ They are stored in the extraction department and collected by personnel from the laboratory as they become available
- ◯ I don't know

Within how many hours/days after blood culture draw do you usually receive a FIRST PRELIMINARY FINDING (e.g., a microscopy finding) of a positive blood culture taken in the emergency department?

Please estimate if no exact data available.

- ○ 0-12h
- ○ 12-24h (1 day)
- ○ 24-48h (1-2 days)
- ○ 48-72h (2-3 days)
- ○ 72-96h (3-4 days)
- ○ more than 4 days
- ○ I don’t have this data and cannot estimate

With regard to the prescription of antibiotics, please indicate clinical practice in your emergency department.

Multiple answer possible.

- ☐ Physicians can prescribe any type of antibiotic
- ☐ Physicians can only prescribe antibiotics which are in stock
- ☐ Physicians need to request authorisation from pharmacy for the prescription of certain antibiotics
- ☐ Physicians need to request authorisation from a consultant
- ☐ Physicians have to consult with the Infectious Disease Department

Questions about sepsis care on the WARDS and/or Intermediate Care Unit (not ICU!).

Please select the kind of wards for which your answers will apply.

Multiple selection possible. If you select only one box, your answer will only apply to this kind of ward!

Please write an email to sepsissurvey@med.uni-greifswald.de ,if you would like to give answers for different kind of wards.
We will sent you an additional survey for this ward.

- [ ] Surgical wards
- [ ] Medical wards
- [ ] Intermediate care unit (without ventilation)
- [ ] Other (please specify)

What is the average number of beds per ward at your hospital?

Beds

How many patients do you treat per month on an average ward?

patients / month

Please estimate the number of sepsis cases per month on an average ward.

[ ] sepsis cases / month

Do you have a protocol or standardized screening tool specifically for the recognition of sepsis on the ward?

- Yes
- No
- I don't know

How often do you screen patients for sepsis on the ward?

- Daily
- Only on demand
- Other (please specify) [ ]
- I don't know

Please select the criteria used to screen patients for sepsis on the ward.

Multiple answers possible.
Please select <u>all</u> applicable criteria!

- [ ] Respiratory rate
- [ ] Temperature
- [ ] Heart rate
- [ ] White blood cells / Leucocytes

- [ ] White blood cells / Leucocytes
- [ ] Mental alteration (e.g. Glasgow Coma Scale)
- [ ] Behavioural change
- [ ] Blood pressure
- [ ] Urine output
- [ ] Lactate
- [ ] Organ dysfunction (e.g. mental status, cardiovascular system, respiration, renal function)
- [ ] qSOFA
- [ ] SOFA
- [ ] SIRS-criteria
- [ ] Early warning scores (e.g. NEWS, MEWS)
- [ ] Procalcitonin (PCT)
- [ ] C-reactive protein (CRP)
- [ ] IL-6
- [ ] Suspected infection
- [ ] Confirmed infection
- [ ] Other (Please describe)

Do you have a protocol, care pathway or bundle specifically for the management of sepsis on the ward?

- Yes
- No
- I don't know

Please choose the actions included in your sepsis protocol, care pathway or bundle on the ward.

Multiple answers possible.

- Measure lactate level
- Obtain blood cultures before administration of antibiotics
- Administer broad-spectrum antibiotics
- Begin rapid administration of 30ml/kg of crystalloid for hypotension or lactate ≥4mmol/L
- Apply vasopressors if patient is hypotensive during or after fluid resuscitation to maintain MAP ≥65mmHg

Do you have a specified time frame for these actions on the ward?

- No
- 1h
- 2h
- 6h
- 12h
- 24h

Please select further actions, that are performed on your ward and the corresponding time frame.

Multiple answers possible.
Please select all applicable criteria!

					Time frame			
	Part of sepsis bundle	Part of sepsis protocol or pathway	Only on demand	Never	within 1-6 hours	within 12 hours	within 24 hours	no time frame
Physical examination	☐	☐	☐	☐	○	○	○	○
Blood testing (white blood cells, coagulation, renal and liver function)	☐	☐	☐	☐	○	○	○	○
SOFA score	☐	☐	☐	☐	○	○	○	○
CT scan for source identification	☐	☐	☐	☐	○	○	○	○
Ultrasound for source identification	☐	☐	☐	☐	○	○	○	○
Chest-X-ray for source identification	☐	☐	☐	☐	○	○	○	○
Microbiological sampling (e.g. urine, swabs, cerebrospinal fluid)	☐	☐	☐	☐	○	○	○	○
Catheterization / urine output measurement	☐	☐	☐	☐	○	○	○	○
Initiation of rapid source control (surgical or	☐	☐	☐	☐	○	○	○	○

interventional)

Central venous line

ScvO2

Arterial line

Passive leg raising or fluid challenge

Cardiac output, stroke volume, pulse pressure measurement

Regular consultations with infectious diseases specialists

Procalcitonin guided anti-infective therapy

MRSA-PCR-testing

Other rapid microbiological testing (please specify)

Other (please specify)

Do you have point-of-care blood gas/lactate analytics on the ward?

- Yes
- No
- I don't know

Do you transfer patients with sepsis from the ward to an intermediate care or intensive care unit?

Multiple answer possible.

- ◯ Yes, we transfer all sepsis cases
- ◯ Yes, but we transfer only patient at higher severity (with shock or need for mechanical ventilation)
- ◯ No, we do not transfer patients with sepsis/septic shock
- ◯ I don't know

Who treats the septic patients on the ward?

- ☐ Ward physician
- ☐ Infectious diseases specialist
- ☐ Antibiotic stewardship team
- ☐ Other (please specify) ____

With regard to the prescription of antibiotics on the ward, please confirm clinical practice in your hospital.

Multiple answer possible.

- ☐ Physicians can prescribe any type of antibiotic
- ☐ Physicians can only prescribe antibiotics which are in stock
- ☐ Physicians need to request authorisation from pharmacy for the prescription of certain antibiotics
- ☐ Physicians need to request authorisation from a consultant
- ☐ Physicians have to consult the Infectious Disease Department

Do you get advice on antibiotic treatment on the ward?

- ◯ Yes
- ◯ No
- ◯ I don't know

Please specify who provides advice on antibiotic treatment on the ward?

- ☐ Antibiotic stewardship team
- ☐ Infectious diseases specialist
- ☐ Microbiologist
- ☐ Other (please specify) ____________

When do you obtain blood cultures on the ward?

Multiple answer possible.

- [ ] They are obtained when the patient has a fever
- [ ] They are obtained when there is shock
- [ ] There are obtained in any case of infection or suspicion of infection
- [ ] They are not routinely obtained

Who takes blood cultures on the ward?

Multiple answers possible.

- [ ] Nursing staff
- [ ] Physicians
- [ ] Specialized phlebotomy staff
- [ ] Depends on the availability of staff

From what site do you collect blood cultures on the ward?

	preferred (primary) collection	first alternative collection	second alternative collection	third alternative collection	never used
Direct venepuncture at two different sites	◯	◯	◯	◯	◯
Direct venepuncture at one site	◯	◯	◯	◯	◯
From new (24h) catheters (arterial or central)	◯	◯	◯	◯	◯
From old (>24h) catheter (arterial or central)	◯	◯	◯	◯	◯
From old (>24h) catheter on suspicion of catheter infection	◯	◯	◯	◯	◯

How many blood cultures do you take when you are suspecting sepsis in a ward patient?

A set consists of 2 vials, one aerobic vial and one anaerobic vial

	always, 100% of the blood culture samplings	> 75% of the blood culture samplings	50-75% of the blood culture samplings	25-50% of the blood culture samplings	less than 25 of the blood culture samplings	never, (0%) of the blood culture samplings
1 set (2 vials)	◯	◯	◯	◯	◯	◯
2 sets (4 vials)	◯	◯	◯	◯	◯	◯
3 sets (6 vials)	◯	◯	◯	◯	◯	◯
4 sets (8 vials)	◯	◯	◯	◯	◯	◯
more than 4 sets	◯	◯	◯	◯	◯	◯

With what priority are blood cultures sent to the laboratory on the ward?

- They are sent immediately after being taken
- They are sent at set times throughout the day (e.g. morning shift, afternoon shift, night shift) irrespective of extraction time
- They are stored in the extraction department and collected by personnel from the laboratory as they become available
- I don't know

How many hours/days after blood culture draw do you usually receive a FIRST PRELIMINARY FINDING (e.g., a microscopy finding) of a positive blood culture obtained on the ward?

Please estimate if no exact data available.

- 0-12h
- 12-24h (1 day)
- 24-48h (1-2 days)
- 48-72h (2-3 days)
- 72-96h (3-4 days)
- more than 4 days
- I don’t have this data and cannot estimate

How long does it usually takes until you get the FINAL MICROBIOLOGICAL RESULT (pathogen identification and antimicrobial susceptibility testing) of a blood culture obtained on the ward?

Please estimate if no exact data available.

- ◯ 1 day
- ◯ 2 days
- ◯ 3 days
- ◯ 4 days
- ◯ 5 days
- ◯ 6 days
- ◯ 7 days
- ◯ more than 7 days

How do you get information about the first finding (e.g., microscopy finding or species identification) of a positive blood culture obtained on the ward?

Multiple answers possible.

- [ ] I have to check and get the information by paper / fax
- [ ] I have to check and get the information via the clinical IT system
- [ ] The laboratory calls me directly
- [ ] I have to call the laboratory
- [ ] The information is given to other staff (e.g. nurses)
- [ ] Other (please describe)

Questions about sepsis care in the INTENSIVE CARE UNIT

Please select the kind of ICU for which you like to answer.

Multiple selection possible. If you select only one box, your answer will only apply to this kind of ICU!

Please write an email to sepsissurvey@med.uni-greifswald.de ,if you would like to give answers for different kind of ICUs.
We will sent you an additional survey for this part.

- ☐ Interdisciplinary
- ☐ Surgical
- ☐ Medical
- ☐ Anaesthesiological
- ☐ Other (please specify)

What measures are available in your ICU?

Multiple selection possible.

- ☐ Mechanical ventilation
- ☐ Blood gas / lactate point-of-care
- ☐ Renal replacement therapy
- ☐ Cardiac output measurement
- ☐ ECMO

How many beds are in your ICU?

Beds [ ]

How many patients do you treat usually per month in the ICU?

[ ] patients / month

Please estimate the number of sepsis cases per month in the ICU.

[ ] sepsis cases / month

Do you have a standardized screening tool specifically for the recognition of sepsis on the ICU?

◯ Yes ◯ No ◯ I don' t know

How often do you screen patients for sepsis in the ICU?

- Daily
- Only on demand
- Other (please specify)
- I don't know

Please select the criteria used to screen patients for sepsis in the ICU.

Multiple answers possible.

- Respiratory rate
- Temperature
- Mental alteration (e.g. Glasgow Coma Scale)
- Behavioural change
- Heart rate
- Blood pressure
- Urine output
- Lactate
- Organ dysfunction (e.g. mental status, cardiovascular system, respiration, renal function)
- qSOFA
- SOFA score
- SIRS-criteria
- Early warning scores (e.g. NEWS, MEWS)

- [ ] Early warning scores (e.g. NEWS, MEWS)
- [ ] White blood cells / Leucocytes
- [ ] Procalcitonin
- [ ] C-reactive protein (CRP)
- [ ] IL-6
- [ ] Suspected infection
- [ ] Confirmed infection
- [ ] Other (Please describe)

Do you have a protocol, care pathway or bundle specifically for the management of sepsis in the ICU?

○ Yes ○ No ○ I don't know

Please choose actions included in your sepsis protocol, care pathway or bundle in the ICU.

Multiple answers possible.

- [ ] Measure lactate level
- [ ] Obtain blood cultures before administration of antibiotics
- [ ] Administer broad-spectrum antibiotics
- [ ] Begin rapid administration of 30ml/kg of crystalloid for hypotension or lactate ≥4mmol/L
- [ ] Apply vasopressors if patient is hypotensive during or after fluid resuscitation to maintain MAP ≥65mmHg

Do you have a specified time frame for these actions in the ICU ?

- ○ No
- ○ 1h
- ○ 2h
- ○ 6h
- ○ 12h
- ○ 24h

Please select further actions, that are performed in the ICU and the corresponding time frame.

Multiple answers possible.
Please select all applicable criteria!

				Time frame			
	Part of						

	Part of sepsis bundle	sepsis protocol or pathway	Only on demand	Never	within 1-6 hours	within 12 hours	within 24 hours	no time frame
Physical examination								
Blood testing (white blood cells, coagulation, renal and liver function)								
SOFA score								
CT scan for source identification								
Ultrasound for source identification								
Chest-X-ray for source identification								
Microbiological sampling (e.g. urine, swabs, cerebrospinal fluid)								
Catheterization / urine output measurement								
Initiation of rapid source control (surgical or interventional)								
Central venous line								
ScvO2								
Arterial line								
Passive leg raising or fluid challenge								

Cardiac output, stroke volume, pulse pressure measurement	☐	☐	☐	☐	○	○	○	○
Regular consultations with infectious diseases specialists	☐	☐	☐	☐	○	○	○	○
Procalcitonin guided anti-infective therapy	☐	☐	☐	☐	○	○	○	○
MRSA-PCR-testing	☐	☐	☐	☐	○	○	○	○
Other rapid microbiological testing (please specify) ____	☐	☐	☐	☐	○	○	○	○
Other (please specify) ____	☐	☐	☐	☐	○	○	○	○

With regard to the prescription of antibiotics in the ICU.

Multiple answer possible.

- ☐ Physicians can prescribe any type of antibiotic
- ☐ Physicians can only prescribe antibiotics which are in stock
- ☐ Physicians need to request authorisation from pharmacy for the prescription of certain antibiotics
- ☐ Physicians need to request authorisation from a consultant
- ☐ Physicians have to consult the Infectious Disease Department

Do you get advice on antibiotic treatment in the ICU?

- ◯ Yes
- ◯ No
- ◯ I don't know

Please specify who provides advice on antibiotic treatment in the ICU?

- ☐ Antibiotic stewardship team
- ☐ Infectious diseases specialist
- ☐ Microbiologist
- ☐ Other (please specify) ____

How do you guide fluid resuscitation in septic shock patients in the ICU?

Multiple answers possible.

- ☐ Blood pressure / heart rate response
- ☐ Urine output
- ☐ Central venous pressure, ScvO2
- ☐ Cardiothoracic ultrasound
- ☐ Pulse pressure variation, cardiac output, stroke volume
- ☐ Lactate clearance / normalization
- ☐ Response to fluid bolus or passive leg raising.
- ☐ We do not use any of these actions

What is your first line catecholamine in the ICU?

- ○ Epinephrine
- ○ Norepinephrine
- ○ Vasopressin
- ○ Other (please describe)

Which fluids do you use for patients with sepsis or septic shock in the ICU?

Multiple answers possible.

- [ ] Saline 0,9%
- [ ] Balanced crystalloids
- [ ] Hydroxyethyl starch
- [ ] Albumin
- [ ] Gelatine
- [ ] Dextran
- [ ] Other (please describe)

When do you obtain blood cultures in the ICU?

Multiple answer possible.

- [ ] They are obtained when the patient has a fever.
- [ ] They are obtained when there is shock
- [ ] There are obtained in any case of infection or suspicion of infection
- [ ] They are not routinely obtained

Who takes the blood cultures in the ICU?

Multiple answers possible.

- [ ] Nursing staff
- [ ] Physicians
- [ ] Specialized phlebotomy staff
- [ ] Depends on the availability of staff

What is your preferred blood culture sample collection site in the ICU?

	preferred (primary) collection	first alternative collection	second alternative collection	third alternative collection	never used
Direct venepuncture at two different sites	○	○	○	○	○
Direct venepuncture at one site	○	○	○	○	○
From new (<24h) catheters (arterial or central)	○	○	○	○	○
From old (>24h) catheter (arterial or central)	○	○	○	○	○
From old (>24h) catheter (arterial or central) on suspicion of catheter infection	○	○	○	○	○

How many blood cultures do you take when sepsis is suspected in the ICU?

A set consists of 2 vials, one aerobic vial and one anaerobic vial

	always, 100% of blood culture samples	more than 75% of blood culture samples	50-75% of blood culture samples	25-50% of blood culture samples	less than 25% of blood culture samples	never, 0% of blood culture samples
1 set (2 vials)	◯	◯	◯	◯	◯	◯
2 sets (4 vials)	◯	◯	◯	◯	◯	◯
3 sets (6 vials)	◯	◯	◯	◯	◯	◯
4 sets (8 vials)	◯	◯	◯	◯	◯	◯
more than 4 sets	◯	◯	◯	◯	◯	◯

With what priority are blood cultures sent to the laboratory obtained in the ICU?

- ◯ They are sent immediately after being taken
- ◯ They are sent at set times throughout the day (e.g. morning shift, afternoon shift, night shift) irrespective of extraction time
- ◯ They are stored in the extraction department and collected by personnel from the laboratory as they become available
- ◯ I don't know

How many hours after blood culture draw do you usually receive a FIRST PRELIMINARY RESULT (e.g., a microscopy finding) of a positive blood culture obtained in the ICU ?

Please estimate if no exact data available.

- ○ 0-12h
- ○ 12-24h (1 day)
- ○ 24-48h (1-2 days)
- ○ 48-72h (2-3 days)
- ○ 72-96h (3-4 days)
- ○ more than 4 days
- ○ I don’t have this data and cannot estimate

How long does it usually take until you get the FINAL MICROBIOLOGICAL RESULT (pathogen identification and antimicrobial susceptibility testing) of a blood culture obtained on the ICU?

Please estimate if no exact data available.

- ◯ 1 day
- ◯ 2 days
- ◯ 3 days
- ◯ 4 days
- ◯ 5 days
- ◯ 6 daye
- ◯ 7 days
- ◯ more than 7 days

How do you get information about the first finding (e.g., microscopy finding or species identification) of a positive blood culture obtained on the ICU ?

Multiple answers possible.

- [ ] I have to check and get the information by paper / fax
- [ ] I have to check and get the information via the clinical IT system
- [ ] The laboratory calls me directly
- [ ] I have to call the laboratory
- [ ] The information is given to other staff (e.g. nurses)
- [ ] Other (please describe)

QUALITY MANAGEMENT and HOSPITAL IMPROVEMENT PROGRAMS at your hospital

Do you have a sepsis training or sepsis quality improvement program?

◯ Yes ◯ No ◯ I don't know

Who is included in your sepsis training or sepsis quality improvement program?

	Emergency department	Ward	Intensive Care Unit	Outpatient clinics	Other areas
Nursing staff at ...	☐	☐	☐	☐	☐
Physicians at ...	☐	☐	☐	☐	☐
Other (e.g. physiotherapists, please specify) ______	☐	☐	☐	☐	☐

Please select the measures which are included in your sepsis training or sepsis quality improvement program.

Multiple answers possible.

- ☐ Meetings and training on a regular basis
- ☐ Case reviews with the team and feedback
- ☐ Information material (e.g. pocket card, poster)
- ☐ External peer-review
- ☐ Other (please describe)

Please select the parameters which are systematically measured in your hospital

Multiple answers possible.

- [ ] Sepsis cases
- [ ] Sepsis mortality
- [ ] Time-to-antibiotics
- [ ] Sepsis bundle compliance (crystalloids, lactate, blood cultures, antibiotics,vasopressors)
- [ ] Severity of sepsis cases
- [ ] Number of blood cultures
- [ ] Other (please describe)
- [ ] We don't measure these parameters

Does your hospital spend extra money on a sepsis program or for specialized sepsis staff?

- ( ) Yes (please describe for what you spend money)
- ( ) No

Are the quality data on contamination rates of blood cultures collected in your hospital?

- ◯ Yes (please specify the blood culture contamination rate in % within the last year) ________
- ◯ No
- ◯ I don't know

Has Covid-19 impacted sepsis management in your area?

- ◯ No
- ◯ Yes (please specify) ________
